# Disulfide-Bridged Cationic Dinuclear Ir(III) Complex with Aggregation-Induced Emission and Glutathione-Consumption Properties for Elevating Photodynamic Therapy

**DOI:** 10.1021/acs.inorgchem.4c04571

**Published:** 2024-12-02

**Authors:** Meijia Huang, Jie Cui, Qi Wu, Shengnan Liu, Dongxia Zhu, Guangzhe Li, Martin R. Bryce, Dong Wang, Ben Zhong Tang

**Affiliations:** † Key Laboratory of Nanobiosensing and Nanobioanalysis at Universities of Jilin Province, Department of Chemistry, Northeast Normal University, 5268 Renmin Street, Changchun, Jilin Province 130024, P. R. China; ‡ Center for AIE Research, College of Materials Science and Engineering, Shenzhen University, Shenzhen 518060, China; § Jilin Provincial Science and Technology Innovation Center of Health Food of Chinese Medicine, Changchun University of Chinese Medicine, Changchun, Jilin Province 130117, P. R. China; ∥ Department of Chemistry, Durham University, Durham DH1 3LE, U.K.; ⊥ School of Science and Engineering, Shenzhen Institute of Aggregate Science and Technology, The Chinese University of Hong Kong, Shenzhen (CUHK-Shenzhen), Guangdong 518172, China

## Abstract

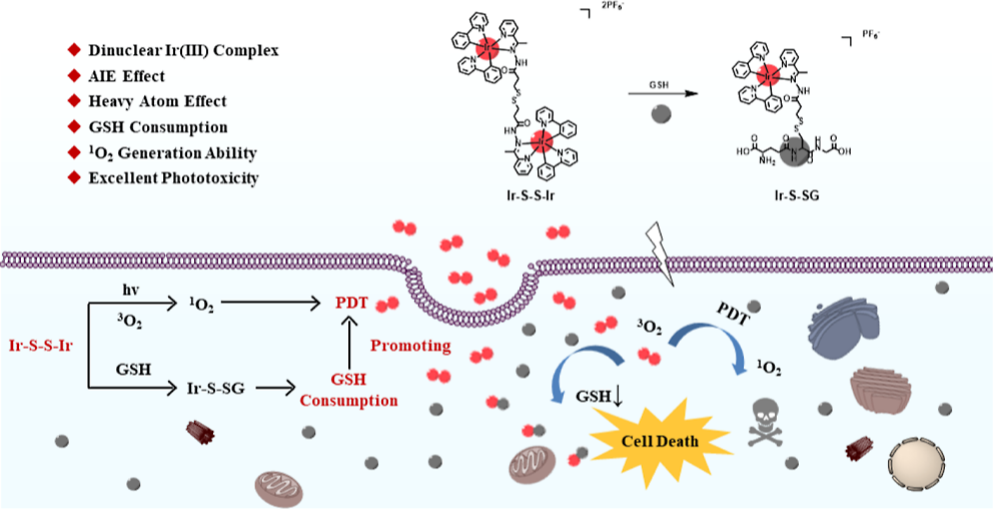

The ability of photosensitizers (PSs) to generate reactive oxygen species (ROS) is crucial for photodynamic therapy (PDT). However, many traditional PSs face the drawbacks that aggregation-caused quenching (ACQ) and highly expressed glutathione (GSH) in the tumor microenvironment seriously limit their ROS generation ability. Herein, we report two cationic dinuclear iridium complexes, **Ir–C–C–Ir** and **Ir–S–S–Ir**, which possess aggregation-induced emission (AIE). **Ir–S–S–Ir** was constructed for GSH consumption by introducing a disulfide linkage between the two auxiliary ligands with imine units. Quantum chemical calculations revealed that **Ir–C–C–Ir** and **Ir–S–S–Ir** possess many degenerate states, which provide more channels for singlet-to-triplet exciton transitions, and then the intersystem crossing rate is increased due to the heavy atom effect of the iridium and sulfur atoms. The ROS production experiments indicated that the singlet oxygen yield of **Ir–S–S–Ir** was 33 times more than that of the ACQ mononuclear iridium complex **Ir–C**. Most importantly, **Ir–S–S–Ir** consumed GSH through a thiol–disulfide exchange reaction, as demonstrated by mass spectrometry and high-performance liquid chromatography. Cell experiments testified that **Ir–S–S–Ir** consumes GSH in tumor cells, possesses good ROS production capacity, and exhibits an extraordinary PDT effect. This is the first report of an AIE dinuclear iridium complex with a GSH-consuming function.

## Abstract 2

A dinuclear iridium complex with both aggregation-induced emission (AIE) and glutathione (GSH)-consuming properties is novel and, to our knowledge, has not been reported until now. The disulfide bridge in the dinuclear structure plays a key role in a thiol−disulfide exchange reaction that consumes GSH. The molecular design provides a new and versatile approach to expand the structures of iridium(III) complexes for PDT against cancer.

## 1 Introduction

Photodynamic therapy (PDT), an emerging clinically approved cancer treatment, has attracted widespread attention due to its limited side effects, noninvasive nature, specificity, and other advantages.^1^ The key factors required for PDT are a photosensitizer (PS), light, and oxygenated substrates.^2^ Upon light activation, PSs reach the excited triplet state (T_1_) through intersystem crossing (ISC). In this state, the PSs can decay back to the singlet ground state by interacting with oxygenated substrates via a process, which can produce cytotoxic reactive oxygen species (ROS), causing oxidation of the cell structures in situ to destroy cancer cells.^3−5^ Therefore, the design of PSs with strong ISC ability that leads to a high level of ROS production remains an urgent problem to be solved.

Compared to many organic molecular PSs, transition metal complex PSs have strong ISC ability, leading to high ROS generation because of their heavy atom effect.^6^ Therefore, utilizing heavy atoms is one of the design strategies used to improve the therapeutic effect of PSs. The iridium(III) atom is a 5d^6^ center, which forms well-known octahedral complexes^7^ whose photophysical properties, such as luminous color and quantum yield, can be regulated by rational adjustment of the type or structure of the ligands.^8^ Ir(III) complexes have other advantages, such as strong ISC ability, good photostability, and efficient cell uptake, leading to a wide range of biological applications.^9−12^ Ir(III) has a higher spin–orbital coupling (SOC) constant (4430 cm^–1^) than other transition metals [Ru(II): 990 cm^–1^, Rh(III): 1425 cm^–1^, Os(II): 3531 cm^–1^, and Pt(II): 4000 cm^–1^] and the strong SOC effect can accelerate the ISC process.^13^ Due to an enhanced heavy atom effect, dinuclear Ir complexes should possess stronger SOC and a narrower singlet–triplet energy gap (Δ*E*_ST_) than mononuclear Ir counterparts, which leads to stronger ISC and higher ROS production capacity.^14,15^ Nevertheless, there are only a few dinuclear or multinuclear Ir complexes reported as PSs due to a lack of reasonable molecular design strategies and complicated synthetic routes.^16−20^

Traditional PSs face the problem that aggregation-caused quenching (ACQ) limits the production of ROS, and overexpressed glutathione (GSH) in the tumor microenvironment reacts with ROS; therefore, ACQ hinders the curative effect of PDT. Due to their rigid, planar, and disc-like structures, traditional PSs, such as boron dipyrromethene (BODIPY), porphyrin, and their derivatives^21−23^ are prone to aggregate in aqueous media due to intermolecular interactions such as π–π stacking. The excited states of these aggregates often decay via nonradiative pathways, immediately leading to undesirable ACQ.^24^ In 2001, Tang’s team rationalized and exploited aggregation-induced emission (AIE).^25,26^ Owing to their unique anti-ACQ effect, AIE molecules have advantages in medical applications such as bioimaging, biomarker detection, comprehensive diagnosis, and treatment.^27,28^ The intramolecular motion of AIE molecules is restricted, which can reduce nonradiative transitions and then increase the ISC rate so that ROS production is enhanced.^29^ Therefore, AIE materials show unique advantages in constructing PSs with outstanding fluorescence and ROS generation properties. AIE-active multinuclear Ir complexes are beneficial for increasing the efficacy of PSs, but they are rare.^30,31^

In tumor cells, large amounts of ROS are mainly produced by the mitochondrial respiratory chain during aerobic metabolism to support their rapid development.^32^ However, excessive ROS can damage DNA, proteins, and lipids, and ultimately lead to cell death.^33^ To combat the damage caused by oxidative stress, the level of the antioxidant system, which is composed of enzymes and nonenzymatic antioxidants in the cell, is upregulated to eliminate excess ROS.^34^ As a prime antioxidant in tumor cells, the concentration of GSH is 2–10 mM, which is much higher than that of normal cells.^35^ Therefore, the effect of PDT, which depends on the ROS generation capacity of PSs, is susceptible to high concentrations of GSH. The elimination of GSH can be achieved by a variety of approaches owing to the diverse pathways of GSH metabolism and the different types of chemical reactions that involve GSH.^36^ The common ways to consume GSH are (i) conversion of GSH to its oxidized disulfide form GSSG; (ii) use of electrophilic reagents to deplete GSH; and (iii) inhibition of GSH synthesis.^37^ Among them, conversion of GSH to the oxidized state through a redox reaction represents one of the most widely used methods for consuming GSH. Metal ions in appropriate oxidation states and disulfides are frequently used as the oxidizing substances.^38^ Disulfide bonds can be cleaved by GSH via a thiol–disulfide exchange reaction, which is a typical redox reaction in tumor cells, resulting in the reversible cleavage and the consumption of GSH at the same time.^39^ Nevertheless, Ir complexes containing a disulfide bond as PSs are still rare.^40,41^ This motivated us to introduce a disulfide bond into an AIE-active dinuclear Ir complex, enabling the consumption of GSH for enhanced PDT.

**Scheme 1:**
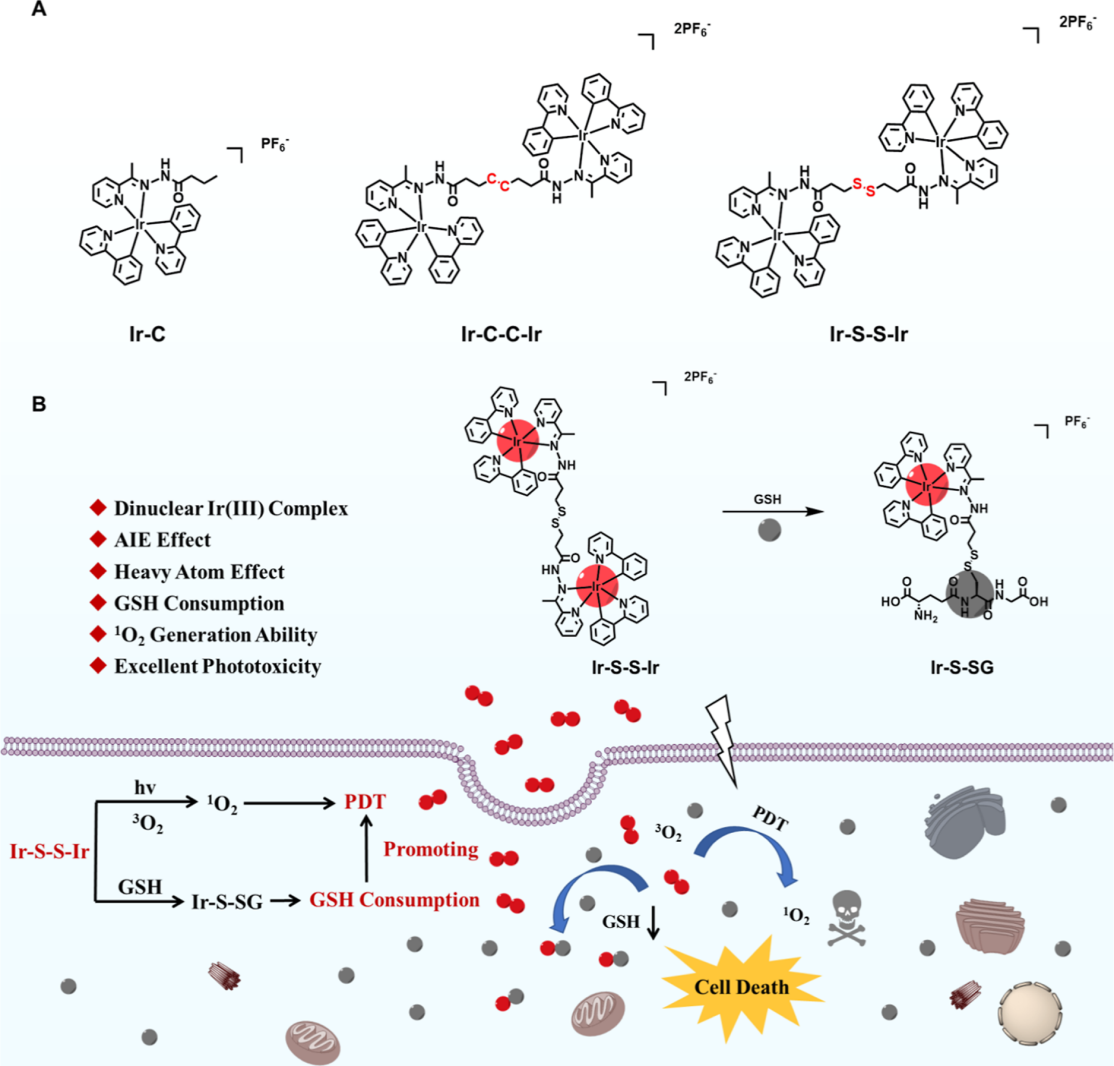
(A) Molecular Structures of Iridium Complexes **Ir–C**, **Ir–C–C–Ir**, and **Ir–S–S–Ir**; (B) Schematic Illustration of **Ir–S–S–Ir** for GSH-Consumed PDT against Cancer

Ir complex PSs with the dual properties of AIE and GSH-consumption represent a new class of PS for PDT. Herein, we obtained the target AIE cationic dinuclear Ir complex, named **Ir–S–S–Ir**, by introducing a disulfide bond into the auxiliary ligands of an acylhydrazone structure (Scheme 1). The model mononuclear complex **Ir–C** showed ACQ, while the dinuclear complexes **Ir–C–C–Ir** and **Ir–S–S–Ir** displayed AIE. Quantum chemical calculations proved that due to the heavy atom effect of the Ir and sulfur atoms, increasing the number of Ir centers effectively improved the ISC ability of **Ir–C–C–Ir** and **Ir–S–S–Ir**, compared with **Ir–C** and then elevated the ROS production. High performance liquid chromatography (HPLC) and mass spectrometry established that **Ir–S–S–Ir** consumed GSH effectively by a thiol–disulfide exchange reaction, whereas **Ir–C–C–Ir** (without the disulfide bond) was unable to react with GSH. Moreover, cell experiments testified that **Ir–S–S–Ir** exhibits stronger ROS production, stronger phototoxicity, and a better PDT effect in 4T1 cells compared with **Ir–C–C–Ir**. This design strategy opens new perspectives for the application of AIE di/multinuclear Ir complexes, endows Ir complexes with the ability to overcome the unfavorable conditions of the tumor microenvironment, and lays the foundation for new functionalization of multinuclear Ir complexes as PSs.

## 2 Materials and Instruments

All solvents and materials were commercially available and used without any further purification. 9,10-Anthracenediyl-bis(methylene) dimalonic acid (ABDA) was purchased from Sigma-Aldrich. Cell counting kit-8 (CCK-8) was purchased from Dojindo Laboratories. Roswell Park Memorial Institute (RPMI-1640) medium, fetal bovine serum, penicillin, and streptomycin were purchased from Gibco. ROS detection kit and cell viability (live dead cell staining) assay kit were purchased from Beyotime.

^1^H nuclear magnetic resonance (NMR) spectra were recorded with a Varian 500 MHz spectrometer and referenced to the residual proton resonances in the solvent. Molecular weights were obtained by using a Bruker autoFlex III mass spectrometer. The UV–vis absorption spectra were measured with a Shimadzu UV-3100 spectrophotometer. The emission spectra, excited-state lifetimes (τ), and photoluminescence quantum yields (PLQYs) were measured with an Edinburgh FLS920 transient fluorescence spectrometer. Transmission electron microscopy (TEM) was recorded on a TECNAI F20 microscope. The diameter and diameter distribution of the nanoparticles were determined by a Malvern Zetasizer Nano instrument for dynamic light scattering (DLS). Confocal laser scanning microscopy (CLSM) images were taken using a ZeissLSM 700 instrument (Zurich, Switzerland). HPLC used Agilent Technologies 1200 Series equipment.

## 3 Results and Discussion

### 3.1 Design, Synthesis, and Photophysical Properties

The complexes were synthesized through conventional methods (Figures S1–S3 in the Supporting Information). Ligands **L1**, **L2**, and **L3** were synthesized through simple Schiff base reactions.^42,43^ Then complexes **Ir–C**, **Ir–C–C–Ir**, and **Ir–S–S–Ir** were synthesized through the reflux of **L1**, **L2**, or **L3** and [Ir(ppy)_2_Cl_2_]_2_ at 80 °C in the dark for 6 h under a N_2_ atmosphere. Characterization data (NMR spectra, mass spectra, and C, H, and N elemental analysis) are given in the Supporting Information. The auxiliary ligand comprised an acylhydrazone structure as a special kind of Schiff base that simultaneously contains imine (–C=N–) and amide (−CONH−) units, in which –C=N– is used for the construction of AIE cationic Ir complexes.^44,45^

**Figure 1:**
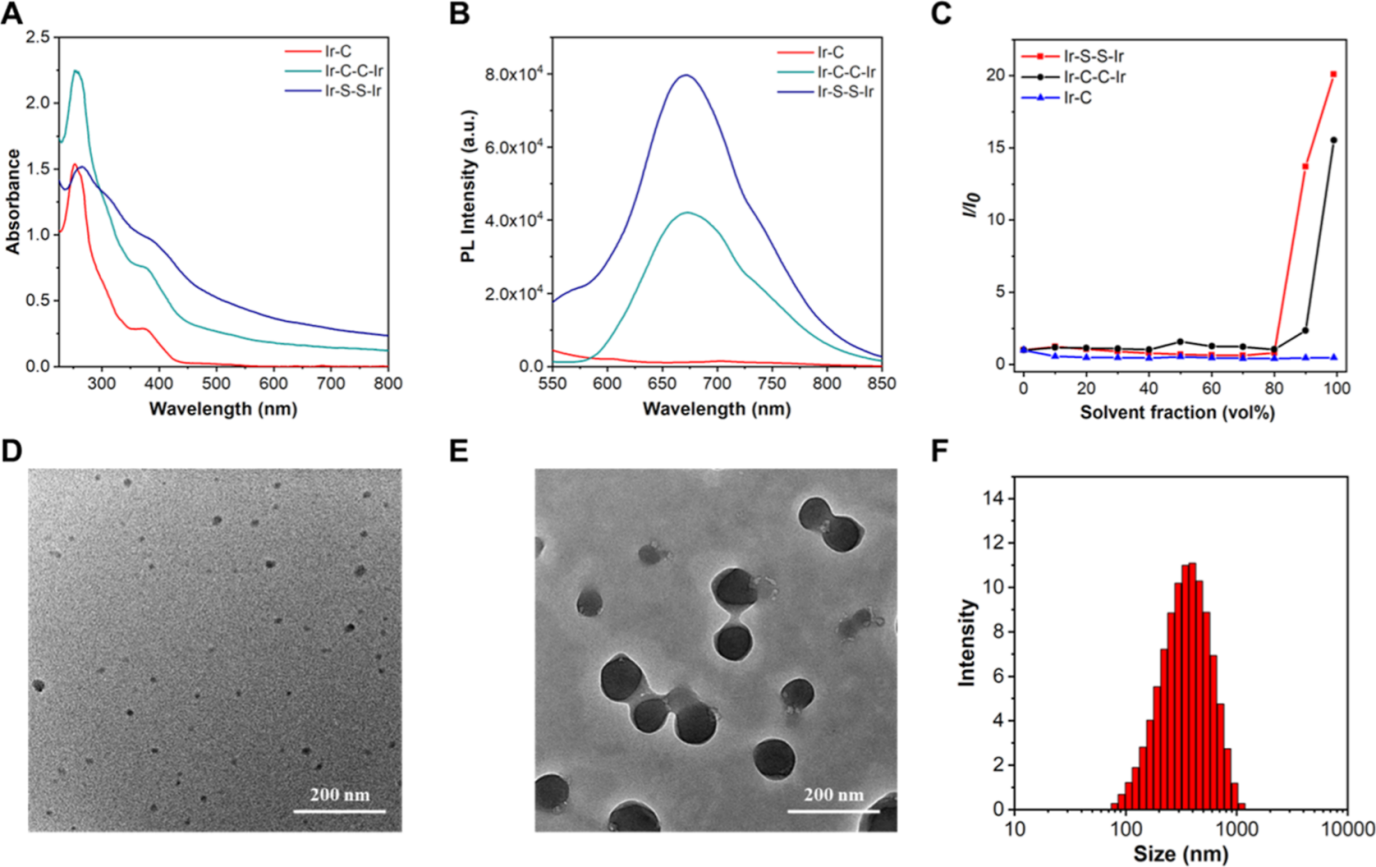
(A) UV–vis absorption spectra and (B) PL spectra in PBS of **Ir–C**, **Ir–C–C–Ir**, and **Ir–S–S–Ir** (5 × 10^–5^ M, pH 7.4). (C) Plots of the relative emission intensity (*I*/*I*_0_) versus solvent fraction. *I*_0_ is the peak values of fluorescence intensities of the Ir complexes in CH_3_CN and *I* is the peak values of fluorescence intensities of the complexes in CH_3_CN/PBS mixtures (pH 7.4). TEM image of nanoaggregates of **Ir–S–S–Ir** formed in CH_3_CN/H_2_O mixtures with 0% water fraction (D) and 99% (E) water fraction. (F) Size distribution of nanoaggregates of **Ir–S–S–Ir** formed in CH_3_CN/H_2_O with 99% water fraction measured by DLS.

The photophysical properties of the complexes were measured by UV–vis absorption and PL spectra. The complexes have a main absorption peak and a shoulder peak, both of which are slightly red-shifted for **Ir–C–C–Ir** and **Ir–S–S–Ir** compared with **Ir–C** (Figure 1A). The strong absorption at 250–350 nm is attributed to the spin-allowed ^1^π–π* transitions on the ligands. The weak absorption bands above 350 nm are due to the spin-forbidden metal-to-ligand charge transfer (^3^MLCT) and ligand-to-ligand charge transfer (^3^LLCT).^46^ The dinuclear complex **Ir–C–C–Ir** has a higher molar absorption coefficient than the mononuclear analogue **Ir–C**. The molar absorption coefficients at 380 nm are 11.9 × 10^3^ M^–1^ cm^–1^ (**Ir–C–C–Ir**) and 6.7 × 10^3^ M^–1^ cm^–1^ (**Ir–C**), respectively, and at 425 nm are 3.6 × 10^3^ M^–1^ cm^–1^ (**Ir–C–C–Ir**) and 1.5 × 10^3^ M^–1^ cm^–1^ (**Ir–C**), respectively. These values for **Ir–C–C–Ir** are therefore 1.78 and 2.4 times those of **Ir–C** at 380 and 425 nm, respectively, ascribed to the additional Ir center in **Ir–C–C–Ir**. Compared with **Ir–C**, **Ir–C–C–Ir** has a stronger light absorption capacity, which should be conducive to PDT. In addition, the three complexes exhibit similar emission spectra in the near-infrared region with λ_max_ at about 720 nm in acetonitrile (Figure 1B). PLQYs and excited state lifetimes (Figure S16) of the three complexes in the solid state are summarized in Table S1. The lifetimes of the dinuclear complexes are 43.1 ns (**Ir–S–S–Ir**) and 38.2 ns (**Ir–C–C–Ir**) compared with 51.9 ns for **Ir–C**. It may be due to the characteristics of AIE that the lifetime of **Ir–S–S–Ir** is slightly greater than that of **Ir–C–C–Ir**.^47,48^ The low quantum yields (Φ_p_ 1.20–1.40) are typical of Ir complexes with near-infrared (700–1700 nm) luminescence.^49,50^

### 3.2 AIE Behavior

To investigate whether the complexes are AIE-active, their emission was measured in a mixed solvent, following established protocols.^51−54^ The complexes are typically poorly soluble in water and toluene. **Ir–C**, **Ir–C–C–Ir**, and **Ir–S–S–Ir** were dissolved in CH_3_CN/phosphate-buffered saline (PBS) mixed solutions (5 × 10^–5^ M) with different water contents (0, 10, 20, 30, 40, 50, 60, 70, 80, 90, 95, and 99%), and then the emission spectra were measured (λ_ex_ = 380 nm, pH 7.4). The emission intensity of **Ir–C** in a CH_3_CN/PBS mixed solution gradually decreased with an increase of water content due to ACQ (Figure S17A). In contrast to **Ir–C**, both **Ir–C–C–Ir** and **Ir–S–S–Ir** in mixed solvent showed an AIE (Figure 1C). **Ir–C–C–Ir** and **Ir–S–S–Ir** exhibited weak emission when the *f*_w_ of the CH_3_CN/PBS mixture varied from 0 to 80% (Figure S17B,C). When *f*_w_ was >90%, strong emission was observed. With the increase of PBS content, the complexes aggregate into nanoparticles in the CH_3_CN/PBS mixture, owing to poor solubility. The molecular structures of the dinuclear complexes **Ir–C–C–Ir** and **Ir–S–S–Ir** are more twisted than that of the mononuclear complex **Ir–C**, which allows strong intermolecular π–π interactions in **Ir–C**. According to previous studies,^44,45^ the AIE properties of **Ir–C–C–Ir** and **Ir–S–S–Ir** are attributed to more supramolecular sites in the auxiliary ligands of the dinuclear complexes, which restrict intramolecular vibrations and rotations in the aggregated state.

UV–vis absorption spectra further demonstrate that the emission enhancement in the mixed solvent originates from molecular aggregation (Figure S18). With an increase in water content in a CH_3_CN/water mixture, the level-off tails clearly appeared in the visible region due to the Mie scattering effect resulting from the aggregated suspensions.^55^ The morphology and size of **Ir–C–C–Ir** and **Ir–S–S–Ir** nanoparticles in the CH_3_CN/water mixed system were measured by TEM and DLS. As shown in Figures S19 and 1D–F, molecular aggregates of the two complexes were formed in the CH_3_CN/water mixture with 99% water. In addition, the average size of the **Ir–C–C–Ir** and **Ir–S–S–Ir** nanoparticles is 294.6 and 302.9 nm, respectively, and the polydispersity index is 0.222 and 0.199, respectively. These results certified that dinuclear complexes **Ir–C–C–Ir** and **Ir–S–S–Ir** are AIE-active, which is conducive to eliminating background interference and favors their application in biology, compared to ACQ mononuclear complex **Ir–C**.

### 3.3 Quantum Chemical Calculations

**Figure 2:**
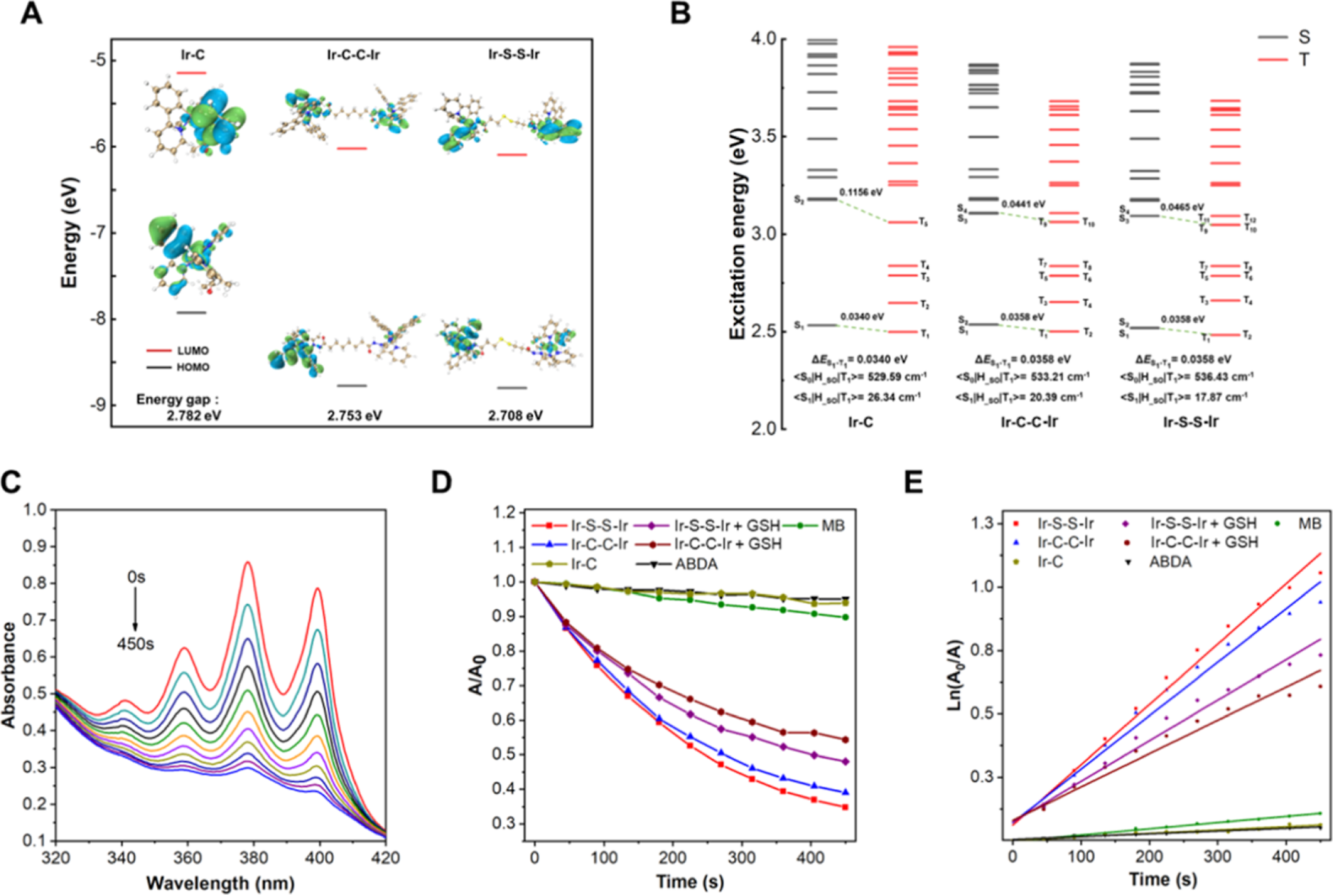
(A) HOMO and LUMO energy level distribution and energy gap of **Ir–C**, **Ir–C–C–Ir**, and **Ir–S–S–Ir**. (B) Singlet and triplet levels of **Ir–C**, **Ir–C–C–Ir**, and **Ir–S–S–Ir** and the values of S_1_–T_1_ and S_0_–T_1_ spiral orbitals. (C) Absorption of ABDA (60 μM) in water in the presence of **Ir–S–S–Ir** (20 μM) under blue light (425 nm, 20 mW cm^–2^) for different times. (D) Plots of the relative absorption intensity (*A*/*A*_0_) at 378 nm versus the irradiation time. *A*_0_ = absorption of ABDA without irradiation. *A* = real-time absorption of ABDA with different irradiation times. (E) Time-dependent ^1^O_2_ generation kinetics under different conditions. *A*_0_ = absorption of ABDA without irradiation. *A* = real-time absorption of ABDA with different irradiation times.

To explore the influence of the number of Ir centers on the energy levels of the excited state of the complexes, quantum chemical calculations were performed on **Ir–C**, **Ir–C–C–Ir**, and **Ir–S–S–Ir** (Figure 2A). It was shown that the highest occupied molecular orbital (HOMO) was mainly distributed on the cyclometalated ĈN ligands, while the lowest unoccupied molecular orbital (LUMO) was mainly distributed on the auxiliary N̂N ligand. Compared with **Ir–C**, the HOMO and LUMO of **Ir–C–C–Ir** and **Ir–S–S–Ir** showed lower energy levels and narrower gaps, which signified a redshift of the absorption wavelength with the increasing number of Ir centers (Figure S20).

To analyze how the number of Ir centers affected the excited states of the complexes, the solvent was calculated at the level of TD-B3LYP/6-31G(d) SMD, DMSO (Figure 2B). Although **Ir–S–S–Ir** and **Ir–C–C–Ir** have higher Δ*E*_ST_, the excitation energy of singlet and triplet states showed that the dinuclear complexes **Ir–C–C–Ir** and **Ir–S–S–Ir** have more degenerate states under the excitation energy of 4 eV, which provides more channels for exciton transition and increases the ISC rate between singlet and triplet states due to the symmetrical structures of **Ir–C–C–Ir** and **Ir–S–S–Ir** (Figure S21). These data indicate that **Ir–C–C–Ir** and **Ir–S–S–Ir** possess higher ISC rates than **Ir–C**, leading to their stronger ROS generation ability.

The MLCT state produced by excitation is the most important intramolecular charge transfer state in Ir complexes.^46^ The excitation energy of the singlet excited state with the maximum oscillator strength of **Ir–C–C–Ir** and **Ir–S–S–Ir** is significantly reduced, and the wavelength is red-shifted by comparing the excited state properties of the complexes (Table S2). According to Figure S22, the contribution of charge transfer from the Ir atom to the ligands (Ir-ligand |q|) in the transition process caused by light absorption of the complex is **Ir–S–S–Ir** > **Ir–C–C–Ir** > **Ir–C**, indicating that the charge transfer of **Ir–S–S–Ir** is the most efficient.

To determine the effects of the configurations of the complexes on the AIE properties and ROS generation capacity, the root-mean-square deviation (RMSD) values of the configurational changes of the S_0_ and T_1_ states after structural relaxation were calculated (Figure S23). **Ir–S–S–Ir** showed the largest RMSD value (2.1125 Å), which means that **Ir–S–S–Ir** possesses the most distorted structure. The calculations also showed that **Ir–C–C–Ir** has a more twisted configuration than **Ir–C** consistent with the number of Ir centers. Twisted structures are beneficial for the complexes to avoid intermolecular π–π stacking in the aggregation state, thereby providing conditions for the generation of AIE,^56^ which inhibits intramolecular motion and promotes the release of excited state energy through ISC, thus promoting the production of ROS.^47^ Therefore, **Ir–S–S–Ir** showed a stronger ROS generation ability than **Ir–C–C–Ir** and **Ir–C**.

To further explore the singlet oxygen generation capacity of **Ir–C–C–Ir** and **Ir–S–S–Ir**, the semiempirical Marcus charge transfer theory was applied. Combining with the CAM-B3LYP group, the Gibbs free energy difference in the energy transfer process was obtained by the energy difference between the optimized ^1^O_2_–S_0_ and T_1_-^3^O_2_ states, which is 0.515 eV (**Ir–C–C–Ir**) and 0.5342 eV (**Ir–S–S–Ir**), respectively. The energy generated by light radiation is sufficient to cross the free energy barrier required for the energy transfer processes, indicating that the complexes have significant advantages in catalyzing the formation of singlet oxygen from triplet oxygen.

### 3.4 Singlet Oxygen Generation Ability

To assess the PDT potential of the complexes, the singlet oxygen production capacity was determined by using ABDA as an indicator. Three control groups were: (1) only complex with irradiation (Figure S24A–C); (2) complex and ABDA without irradiation (Figure S24D–F); and (3) only ABDA with irradiation (Figure S24G). The absorption intensity of the three control groups did not change significantly within 450 s under respective conditions. This showed that the complexes have good photostability, and singlet oxygen would not be produced by the complexes in the absence of light. When the mixed solution of complexes and ABDA was illuminated, the absorption intensity of ABDA at 378 nm declined significantly in the solution containing **Ir–S–S–Ir** or **Ir–C–C–Ir** (Figures 2C and S24H), while the absorption strength decreased only slightly in the solution containing **Ir–C** (Figure S24I). These data proved that **Ir–S–S–Ir** and **Ir–C–C–Ir** could produce singlet oxygen rapidly with irradiation, whereas **Ir–C** could generate only a small amount of singlet oxygen.

As shown in Figure 2D,E, the singlet oxygen generation of the complexes conforms to a first-order kinetic equation. Methylene blue (MB) was used as the reference in the experiment. The slope follows the order: **Ir–S–S–Ir** (0.00238) > **Ir–C–C–Ir** (0.00211) > MB (0.00025) > **Ir–C** (0.00013) (Table S3). The slope of **Ir–C–C–Ir** is 16.23 times that of **Ir–C**, indicating that **Ir–C–C–Ir** exhibits a much stronger singlet oxygen production capacity than **Ir–C**. According to the literature,^57^ singlet oxygen yields of the complexes were calculated from the following formula, taking MB (singlet oxygen yield of 52%) as the reference.

The singlet oxygen yield of **Ir–S–S–Ir**, **Ir–C–C–Ir**, and **Ir–C** is 76.7, 31.5, and 2.3%, respectively, which means that the **Ir–S–S–Ir** yield is about 2.4 times that of **Ir–C–C–Ir**, and **Ir–C–C–Ir** is about 13.7 times that of **Ir–C**. Noteworthy, the slopes for **Ir–S–S–Ir** and **Ir–C–C–Ir** are similar, which indicates that the dinuclear complexes produce singlet oxygen at a similar rate under 425 nm irradiation. However, the absorbance of **Ir–S–S–Ir** at 425 nm is 0.0236 (Figure S24C), which is smaller than that of **Ir–C–C–Ir** (0.0503) (Figure S24B). The data indicated that **Ir–S–S–Ir** absorbs less energy at 425 nm than does **Ir–C–C–Ir**. According to the formula for calculating singlet oxygen yield, **Ir–S–S–Ir** has weaker absorption and a faster singlet oxygen generation rate under 425 nm irradiation, leading to greater singlet oxygen yield. The above results indicate that increasing the number of Ir centers and introducing sulfur atoms with an accompanying heavy atom effect improves the probability of ISC and promotes the ROS production ability, which is consistent with the quantum chemical calculations. Based on the combination of computational results and the experimental data (comparison of **Ir–C** with **Ir–C–C–Ir** and **Ir–C–C–Ir** with **Ir–S–S–Ir**), it can be concluded that dinuclear Ir complexes have great potential as PSs in PDT therapy.

To determine the effect of the high content of GSH in tumor cells on the singlet oxygen generation capacity of **Ir–C–C–Ir** and **Ir–S–S–Ir**, 10 mM GSH was added to the test solution to simulate the tumor microenvironment. **Ir–S–S–Ir** and **Ir–C–C–Ir** still possessed the ability to produce singlet oxygen in the environment with a high GSH content (Figure S24J,K). The singlet oxygen production rates decreased due to the reducibility of GSH, which can reduce the ROS produced by the complexes under irradiation (Figure 2D,E). This data indicated that a high content of GSH in the tumor microenvironment can adversely affect the PDT efficacy of PSs. The singlet oxygen generation capacity of **Ir–C–C–Ir** and **Ir–S–S–Ir** still conforms to the first-order kinetic equation with the addition of GSH. The slope order is **Ir–S–S–Ir** (0.0016) > **Ir–C–C–Ir** (0.00131). Moreover, the singlet oxygen yield of **Ir–S–S–Ir** decreased to 46.9% with GSH, which is 61.1% of that without GSH. The singlet oxygen yield of **Ir–C–C–Ir** declined to 15.7%, which is only 49.8% of that without GSH. Importantly, the above results indicated: (i) that the singlet oxygen production capacity of **Ir–S–S–Ir** is less affected by GSH than that of **Ir–C–C–Ir** due to **Ir–S–S–Ir** consuming GSH and (ii) **Ir–S–S–Ir** can weaken the adverse effect of GSH on PDT.

### 3.5 GSH Consumption in Solution

#### 3.5.1 Analysis of Structural Changes in the Reaction of **Ir–S–S–Ir** with GSH

**Figure 3:**
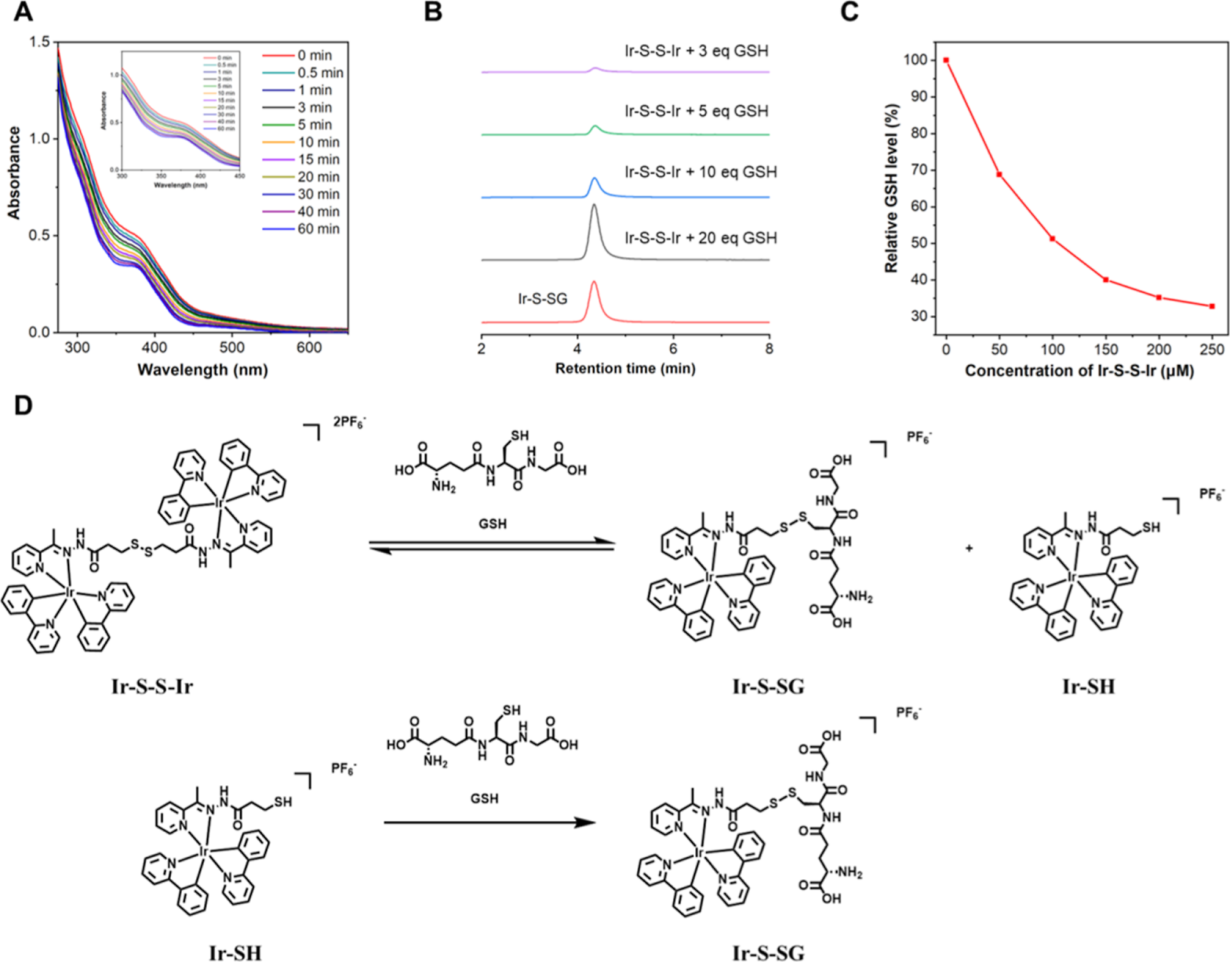
(A) UV–vis absorption spectra of **Ir–S–S–Ir** (30 μM) mixed with GSH (2 mM) in aqueous solution for different times. (B) HPLC of **Ir–S–S–Ir** reaction with GSH. (C) Concentration-dependent curve of **Ir–S–S–Ir** interaction with GSH in PBS (pH 7.4). (D) Proposed mechanism of **Ir–S–S–Ir** reaction with GSH.

To explore the thiol–disulfide exchange reaction between the disulfide bond in **Ir–S–S–Ir** and highly expressed GSH in the tumor microenvironment, the reaction of **Ir–S–S–Ir** with GSH was monitored by UV–vis absorption spectroscopy (Figure 3A). The absorption strength of **Ir–S–S–Ir** decreased gradually within 60 min after mixing with GSH, while that of **Ir–C–C–Ir** remained unchanged (Figure S25). These results confirmed that **Ir–C–C–Ir** is unable to react with GSH owing to the absence of a disulfide bond, whereas **Ir–S–S–Ir** undergoes the thiol–disulfide exchange reaction, which destroys the **Ir–S–S–Ir** structure and, consequently, the absorption strength is decreased.

Mass spectrometry was used to investigate the structure of the product after the reaction of **Ir–S–S–Ir** with GSH. According to the established reaction mechanism,^36,37^ GSH attacks the disulfide bond in **Ir–S–S–Ir** to generate mononuclear complexes Ir–S–SG and Ir-SH, through a nucleophilic substitution reaction (S_N_2) (Figure 3D). In this reversible reaction, excessive GSH is not conducive to the reverse reaction, resulting in **Ir–S–S–Ir** remaining in the system, which can be used for the generation of singlet oxygen. At the same time, the sulfhydryl (SH) group in Ir-SH will quickly form a new disulfide bond with GSH.^58^ The theoretical *m*/*z* value of the product Ir–S–SG is 1029.2404, and a mass spectrum peak was found at *m*/*z* 1029.2995 in the postreaction mixture, which is consistent with the theoretical value (Figure S26). Therefore, the product can be clearly identified as Ir–S–SG.

The reaction of **Ir–S–S–Ir** with GSH was also analyzed by HPLC (Figure 3B). The peak area from the product increased gradually with the increasing concentration of GSH while the concentration of **Ir–S–S–Ir** and the reaction time remained the same. The retention time of the product was consistent with that of Ir–S–SG under the same chromatographic conditions, which confirmed that the reaction product was Ir–S–SG. The results proved that the thiol–disulfide exchange reaction occurs between **Ir–S–S–Ir** and GSH. Therefore, it is expected that the PDT efficiency of **Ir–S–S–Ir** can be improved through the consumption of GSH.

#### 3.5.2 GSH Consumption Capacity Test in Solution

As reported in previous literature,^59^ DTNB [5,5′dithio-bis(2-nitrobenzoic acid)] colorimetry was used to determine the consumption capacity of **Ir–S–S–Ir** on GSH, which is concentration-dependent with the same concentration of GSH (1.3 mM) (Figure 3C). When the concentration of **Ir–S–S–Ir** reached 250 μM, the consumption ratio of GSH was close to 30%, indicating that **Ir–S–S–Ir** displays a good GSH consumption capacity.

### 3.6 Cytotoxicity of the Complexes against 4T1 Cells

**Figure 4:**
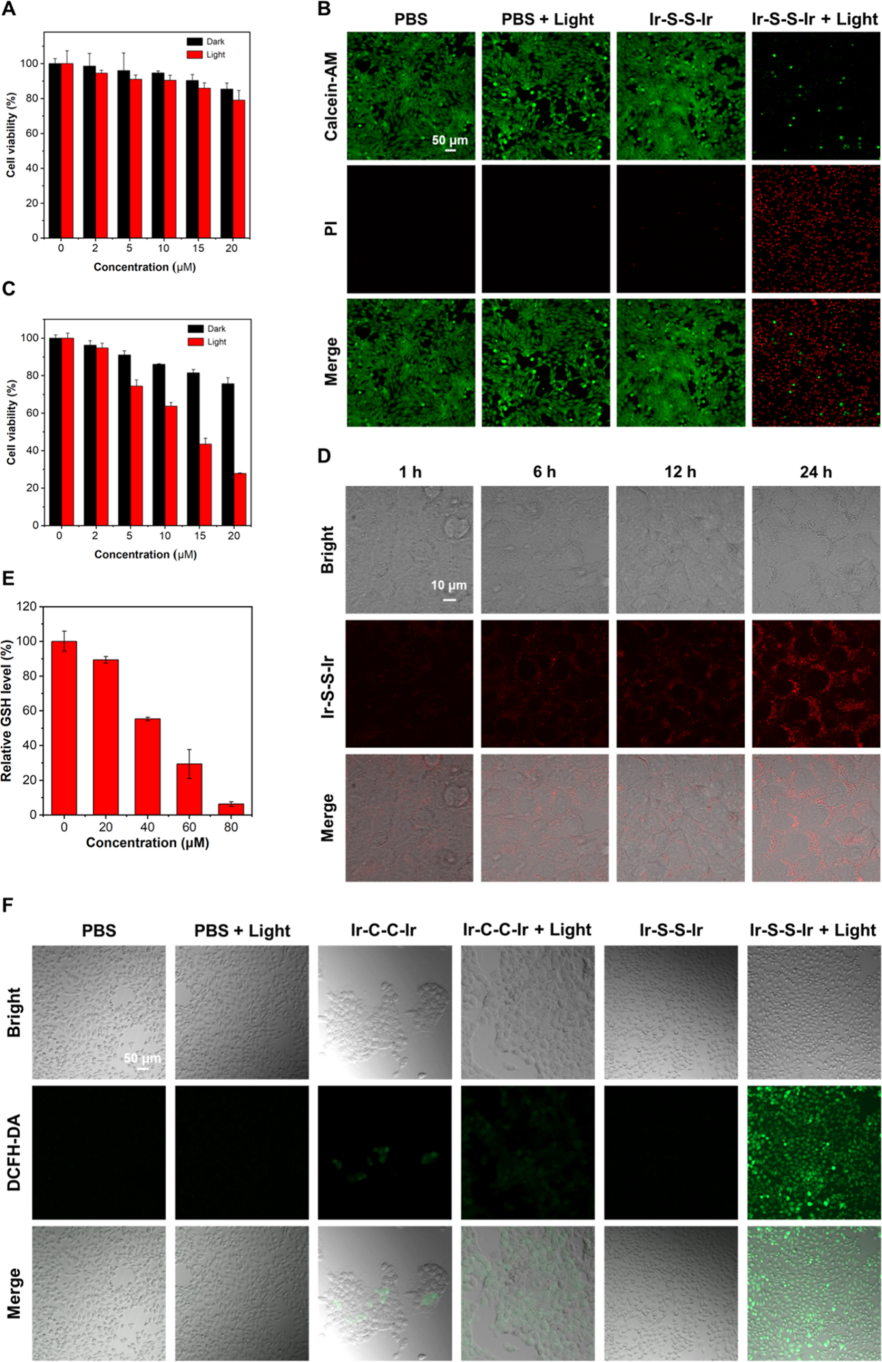
Cell viability of (A) **Ir–C–C–Ir** and (C) **Ir–S–S–Ir** against 4T1 cells under dark and under light (400–800 nm, 20 mW cm^–2^, 20 min). (B) Fluorescence images of living/dead 4T1 cells incubated with **Ir–S–S–Ir** (20 μM) under white light irradiation (400–800 nm, 20 mW cm^–2^) and dark conditions. (D) CLSM images after **Ir–S–S–Ir** was incubated with 4T1 cells for 1, 6, 12, and 24 h. (E) Changes of GSH concentration in 4T1 cells treated with different concentrations of **Ir–S–S–Ir**. (F) Generation of intracellular ROS mediated by **Ir–C–C–Ir** (50 μM) and **Ir–S–S–Ir** (20 μM) upon irradiation (400–800 nm, 20 mW cm^–2^, 20 min) as indicated by the fluorescence of the oxidized state DCF.

The above data demonstrate that **Ir–C–C–Ir** and **Ir–S–S–Ir** have greater ROS generation capability compared with **Ir–C**. Therefore, the viability of 4T1 cells (which are breast cancer cell line derived from the mammary gland tissue of a mouse) incubated with **Ir–C–C–Ir** and **Ir–S–S–Ir** was evaluated by CCK-8 assays (Figure 4A,C). When the concentration of **Ir–S–S–Ir** reached 20 μM, the cell viability was only about 30%, indicating that **Ir–S–S–Ir** possesses good phototoxicity while the cell viability remained at 80% with the same concentration of **Ir–C–C–Ir** under the irradiation of white light (400–800 nm, 20 mW cm^–2^). Noteworthy, the increase of **Ir–S–S–Ir** concentration also caused a slight decrease of cell viability in the dark due to the consumption of GSH by the disulfide bond. The consumption of GSH could affect the cell activity, so that **Ir–S–S–Ir** showed mild cell killing ability under dark conditions.

### 3.7 Live/Dead Cell Staining Experimental Study

To determine the killing of tumor cells by the complexes after illumination, 4T1 cells were incubated under different conditions and costained by Calcein-AM and propidium iodide. As shown in Figure 4B, most of the tumor cells emit red fluorescence after **Ir–S–S–Ir** incubation and illumination, while only a few produce green fluorescence, which indicated that most tumor cells were killed. In the dark conditions, the tumor cells emitted green fluorescence, and a small amount of red fluorescence appeared, testifying that only a few cells died. These results proved that **Ir–S–S–Ir** showed slight dark toxicity and high phototoxicity, which is consistent with the cytotoxicity against 4T1 cells. However, only a few of the tumor cells incubated with **Ir–C–C–Ir** were killed under white light irradiation, which means that **Ir–C–C–Ir** exhibits poor phototoxicity (Figure S28). It is therefore demonstrated that **Ir–S–S–Ir** has good potential as a PS in PDT therapy compared to **Ir–C–C–Ir**.

### 3.8 Intracellular Photoinduced ROS Generation Ability of the Complexes

To further explore the application of the complexes in cells, dichlorodihydrofluorescein diacetate (DCFH-DA) was used as an indicator of the ROS production capacity of the complexes in 4T1 cells. As shown in Figure 4F, confocal imaging revealed negligible fluorescence in the control, with or without light irradiation, after cells were treated with **Ir–S–S–Ir** and **Ir–C–C–Ir** in dark conditions. Enhanced green fluorescence from the oxidized state DCF was observed after white light irradiation of cells incubated with **Ir–S–S–Ir**, illustrating the excellent ROS generation ability of **Ir–S–S–Ir**. In contrast, the green fluorescence was weak after irradiation of cells incubated with **Ir–C–C–Ir**, demonstrating poor ROS generation from **Ir–C–C–Ir**. These results indicate that **Ir–S–S–Ir** has good application potential as a PS in PDT.

### 3.9 Intracellular GSH Content Test

The ability of **Ir–S–S–Ir** to consume GSH in cancer cells was tested with a reduced GSH detection kit. With increasing **Ir–S–S–Ir** concentration, the GSH content in 4T1 cells gradually decreased (Figure 4E). When the concentration of **Ir–S–S–Ir** reached 40 μM, the GSH content decreased by half; when the concentration reached 80 μM, the GSH content was less than 10%. These results certified that **Ir–S–S–Ir** possesses an outstanding ability to consume GSH in cancer cells. In conclusion, **Ir–S–S–Ir** displays excellent ROS production ability and also consumes GSH in cancer cells, which can overcome the unfavorable conditions of high content of GSH in cancer cells, and **Ir–S–S–Ir** can be used as a dual-function PS in PDT.

### 3.10 Cellular Uptake

To determine the uptake of the complexes in tumor cells, the intracellular luminescence of the complexes was investigated by a CLSM. The phosphorescence signal generated in the cells gradually increased with the extension of the culture time, indicating that the cellular uptake was time-dependent (Figure 4D). After **Ir–S–S–Ir** was incubated with 4T1 cells for 24 h, the red luminescence intensity of **Ir–S–S–Ir** reached a maximum, indicating that the cells had the highest uptake of **Ir–S–S–Ir** at this time. The red luminescence intensity of **Ir–C–C–Ir** reached its maximum after the cells were incubated for 12 h (Figure S29). These results indicate that **Ir–S–S–Ir** can be effectively taken up by tumor cells, which is conducive to its killing effect on tumor cells.

## 4 Conclusions

Ir complex PSs with the dual properties of AIE and GSH-consumption represent a new class of PS for PDT. Two AIE-active cationic dinuclear Ir complexes, **Ir–C–C–Ir** and **Ir–S–S–Ir**, were synthesized by utilizing the imine units in the auxiliary ligands. A multifunctional PS with GSH-consuming ability was achieved with **Ir–S–S–Ir**, thereby enriching the application of multinuclear Ir complexes in PDT. Quantum chemistry calculations proved that the dinuclear complexes, **Ir–C–C–Ir** and **Ir–S–S–Ir**, possess stronger ISC capacity compared to the model mononuclear complex **Ir–C** because of the heavy atom effect of the additional Ir and sulfur atoms. Experiments indicated that **Ir–C–C–Ir** and **Ir–S–S–Ir** display AIE and much enhanced ROS production ability compared to **Ir–C** that shows ACQ. Moreover, **Ir–S–S–Ir** consumes GSH through a thiol–disulfide exchange reaction in solution certified by mass spectrometry and HPLC data. Cell experiments using 4T1 cells established that **Ir–S–S–Ir** consumes GSH and possesses an excellent ROS production capacity. In addition, **Ir–S–S–Ir** showed superior phototoxicity and cell uptake ability and exhibited an outstanding PDT effect. This work describes **Ir–S–S–Ir** as the first AIE-active dinuclear Ir complex with a GSH-consuming function, providing a new strategy for the application of Ir complexes in PDT. It is now clear that di/multinuclear Ir complex PSs offer good prospects in the clinical applications of PDT.

## Data Availability

The data associated with this article is available in the manuscript and Supporting Information files. Additional data will be made available on request.

## Supporting Information Available

Details of synthesis and characterization including NMR spectra of all compounds; additional calculation results; and detailed cell uptake and in vitro experimental results (PDF)
