# Supplementary material for: Disulfide-Bridged Cationic Dinuclear Ir(III) Complex with Aggregation-Induced Emission and Glutathione-Consumption Properties for Elevating Photodynamic Therapy
Source: Inorg Chem. 2024 Dec 2;63(50):24030–40. doi: 10.1021/acs.inorgchem.4c04571 (PMC11653252; doi:10.1021/acs.inorgchem.4c04571)
Supplement: Supplementary file 1 — ic4c04571_si_001.pdf [file ic4c04571_si_001.pdf]

Supporting Information

Disulfide-Bridged Cationic Dinuclear Ir(III) Complex with Aggregation Induced Emission and Glutathione-Consumption Properties for Elevating Photodynamic Therapy

Meijia Huang [‡a], *Jie Cui* [‡b], *Qi Wu* [‡a], *Shengnan Liu* [a], *Dongxia Zhu* [a*], *Guangzhe Li* [c*], *Martin R. Bryce* [d*], *Dong Wang* [b*], *Ben Zhong Tang* [b,e*]

[a] Key Laboratory of Nanobiosensing and Nanobioanalysis at Universities of Jilin Province, Department of Chemistry, Northeast Normal University, 5268 Renmin Street, Changchun, Jilin Province 130024, P.R. China.

*E-mail: zhudx047@nenu.edu.cn

[b] Dr. J. Cui and Prof. D. Wang

Center for AIE Research, College of Materials Science and Engineering, Shenzhen University, Shenzhen 518060, China.

*E-mail: wangd@szu.edu.cn

[c] Prof. G. Li

Jilin Provincial Science and Technology Innovation Center of Health Food of Chinese Medicine, Changchun University of Chinese Medicine, Changchun, Jilin Province 130117, P. R. China.

*E-mail: 1993008106@qq.com

[d] Prof. Martin R. Bryce

Department of Chemistry, Durham University, Durham, DH1 3LE, UK.

*E-mail: m.r.bryce@durham.ac.uk

[e] Prof. B. Z. Tang

School of Science and Engineering, Shenzhen Institute of Aggregate Science and Technology, The Chinese University of Hong Kong, Shenzhen (CUHK-Shenzhen), Guangdong 518172, China

*E-mail: tangbenz@cuhk.edu.cn

‡ These authors contributed equally to this work.

S1 Experimental

S1.1 Synthesis

S1.1.1 Synthesis of ***L1***

A mixture of butyric acid hydrazide (0.1021 g, 1 mmol) and 2-acetylpyridine (0.3634 g, 3 mmol) was dissolved in ethanol (15 mL). Then 4 drops of ice-cold acetic acid were added to the mixture, and the mixture was refluxed at 80 °C for 8 h. The solvent was removed by spin steaming after cooling to room temperature. The product was purified by silica gel column chromatography with dichloromethane/ethyl acetate (10/2 v/v) as eluting solvent. The product was a white solid with a yield of 50%. ^{1}H NMR (600 MHz, Chloroform-*d*) δ 8.62 (s, 1H), 8.59 (d, *J* = 4.6 Hz, 1H), 8.05 (d, *J* = 8.0 Hz, 1H), 7.72 (d, *J* = 7.7 Hz, 1H), 2.78 (t, *J* = 7.5 Hz, 2H), 2.35 (s, 3H), 1.78 (q, *J* = 7.4 Hz, 2H), 1.04 (t, *J* = 7.4 Hz, 3H) (Figure S4).

S1.1.2 Synthesis of ***L2*** *and* ***L3***

Dimethyl suberate (0.8495 g, 4.2 mmol) and dimethyl 3,3'-disulfanediyldipropionate (1.0 g, 4.2 mmol) were dissolved in methanol (25 mL) and then hydrazine 80% hydrate (5 mL) was added to the mixture which was then stirred at room temperature for 12 h. As the reaction progressed, a white precipitate appeared. Then the precipitate was filtered, washed with methanol many times and dried. The intermediate product **1** was obtained with a yield of 80%. ^{1}H NMR (600 MHz, DMSO-d_6) δ 8.91 (s, 2H), 4.15 (s, 4H), 2.00 (t, *J* = 7.4 Hz, 4H), 1.50-1.44 (m, 4H), 1.22 (p, *J* = 3.8 Hz, 4H). The intermediate product **2** was similarly obtained with a yield of 85%. ^{1}H NMR (600 MHz, DMSO-d_6) δ 9.08 (s, 2H), 4.22 (s, 4H), 2.91 (t, *J* = 7.2 Hz, 4H), 2.42 (t, *J* = 7.2 Hz, 4H).

The intermediate product **1** (0.1011 g, 0.5 mmol) or **2** (0.1192 g, 0.5 mmol) was mixed with 2-acetylpyridine (0.3633 g, 3 mmol). Then the mixture was dissolved in ethanol (10 mL), and 4 drops of

ice-cold acetic acid were added. The reaction mixture was refluxed at 80 °C for 16 h. With the increase of temperature, **1** and **2** gradually dissolved and then the product precipitated. After cooling to room temperature, the precipitate was filtered, collected, washed with ethanol many times and dried. **L2** was obtained as white solid with a yield of 75%. ^{1}H NMR (600 MHz, DMSO-d_6) δ 10.53-10.42 (m, 2H), 8.65-8.49 (m, 2H), 8.02 (t, J = 8.0 Hz, 2H), 7.82 (q, J = 7.4 Hz, 2H), 7.45-7.29 (m, 2H), 2.70 (s, 3H), 2.32 (d, J = 13.3 Hz, 6H), 1.62 (s, 4H), 1.39 (s, 4H) (Figure S5). **L3** was obtained as white solid with a yield of 75%. ^{1}H NMR (400 MHz, DMSO-d_6) δ 10.75-10.58 (m, 2H), 8.57 (d, J = 4.7 Hz, 2H), 8.02 (d, J = 8.2 Hz, 2H), 7.82 (d, J = 5.9 Hz, 2H), 7.42-7.31 (m, 2H), 3.14 (t, J = 6.4 Hz, 2H), 3.05 (d, J = 6.1 Hz, 4H), 2.86-2.65 (m, 2H), 2.36-2.29 (m, 6H) (Figure S6).

S1.1.3 Synthesis of ***Ir-C*****,** ***Ir-C-C-Ir*** *and* ***Ir-S-S-Ir***

The cyclometalated chloride-bridged dimer $[Ir(ppy)_2Cl_2]_2$ was synthesized using the classical method.[1,2] 2-Phenylpyridine (0.388 g, 2.5 mmol) and $IrCl_3·H_2O$ (0.3571 g, 1 mmol) were dissolved in a mixture of ethylene glycol ether and water (3:1, v/v) (40 mL) under N_2 atmosphere at 120 °C and refluxed for 24 h. After cooling to room temperature, more water was added into the mixture to acquire a precipitate which was filtered and washed with water and ethanol for many times. The remaining product which was yellow solid was not purified and used directly in the next step.

The auxiliary ligand **L1** (0.0411 g, 0.2 mmol), **L2** (0.041 g, 0.1 mmol) or **L3** (0.044 g, 0.1 mmol) was dissolved in a mixture of dichloromethane (15 mL) and methanol (15 mL). $[Ir(ppy)_2Cl_2]_2$ (0.1072 g, 0.1 mmol) was added and the mixture was refluxed at 80 °C in the dark for 6 h under N_2 atmosphere. After cooling to room temperature, excess KPF_6 was added and the mixture was stirred at room temperature for 30 min. The suspension was then filtered and the solvent was removed under vacuum. The crude product was purified by silica gel column chromatography.

Ir-C was purified by silica gel column chromatography with dichloromethane/methanol (200/1 v/v) as eluting solvent. The product was a red solid with a yield of 90%. ^{1}H NMR (600 MHz, DMSO-d_6) δ 10.49 (s, 1H), 8.64 (s, 1H), 8.52 (d, *J* = 8.0 Hz, 1H), 8.27 (t, *J* = 7.9 Hz, 1H), 8.23 (d, *J* = 8.2 Hz, 1H), 8.18 (d, *J* = 8.1 Hz, 1H), 7.96 (t, *J* = 7.8 Hz, 2H), 7.88 (d, *J* = 7.7 Hz, 1H), 7.81-7.78 (m, 2H), 7.76-7.73 (m, 1H), 7.43 (s, 1H), 7.29 (t, *J* = 6.5 Hz, 1H), 7.19 (t, *J* = 6.4 Hz, 1H), 6.99 (t, *J* = 7.4 Hz, 1H), 6.93 (t, *J* = 7.4 Hz, 1H), 6.85 (t, *J* = 7.1 Hz, 1H), 6.78 (t, *J* = 7.4 Hz, 1H), 6.14 (d, *J* = 7.6 Hz, 1H), 6.04 (d, *J* = 7.5 Hz, 1H), 2.62 (s, 3H), 1.70-1.60 (m, 2H), 1.13 (p, *J* = 7.4 Hz, 2H), 0.67 (t, *J* = 7.4 Hz, 3H) (Figure S7). ^{13}C NMR (126 MHz, DMSO-d_6) δ 177.66, 167.67, 154.45, 152.19, 150.43, 150.34, 149.49, 144.55, 144.39, 140.02, 139.10, 139.02, 132.65, 131.31, 130.77, 130.42, 129.83, 129.43, 125.38, 124.77, 124.07, 123.87, 122.80, 122.24, 120.13, 120.07, 35.23, 18.43, 14.06 (Figure S8). ESI-MS: [m/z] = 706.2163 (M^+) (Figure S9). Calcd. for $C_{33}H_{31}F_6IrN_5OP$: C 46.59 H 3.67, N 8.23. Found C 46.62, H 3.70, N 8.26.

Ir-C-C-Ir was purified by silica gel column chromatography with dichloromethane/methanol (100/6 v/v) as eluting solvent. The product was a red solid with a yield of 80%. ^{1}H NMR (500 MHz, DMSO-d_6) δ 10.52 (s, 2H), 8.67 (s, 2H), 8.53 (d, *J* = 8.0 Hz, 2H), 8.28 (t, *J* = 7.2 Hz, 2H), 8.24 (d, *J* = 8.1 Hz, 2H), 8.16 (s, 2H), 7.97 (t, *J* = 7.6 Hz, 4H), 7.89 (d, *J* = 7.8 Hz, 2H), 7.78 (dt, *J* = 13.4, 5.4 Hz, 6H), 7.45 (t, *J* = 7.6 Hz, 2H), 7.31-7.27 (m, 2H), 7.20 (s, 2H), 6.99 (t, *J* = 7.4 Hz, 2H), 6.88 (dt, *J* = 25.0, 7.3 Hz, 4H), 6.77 (s, 2H), 6.16 (dd, *J* = 7.1, 4.2 Hz, 2H), 6.03 (d, *J* = 7.5 Hz, 2H), 2.63 (s, 6H), 1.66 (s, 4H), 1.02 (s, 4H), 0.93 (s, 4H) (Figure S10). ^{13}C NMR (126 MHz, Methylene Chloride-d_2) δ 173.96, 168.78, 167.14, 166.99, 154.79, 150.92, 150.78, 149.17, 143.90, 139.40, 138.73, 138.36, 132.13, 131.54, 130.68, 130.49, 129.67, 128.52, 124.89, 124.81, 123.90, 123.73, 123.36, 122.99, 119.73, 119.63, 33.65, 29.68, 28.27, 24.29, 17.66 (Figure S11). ESI-MS: [m/z] = 706.2163 (M^{2+}) (calcd:

706.2163) (Figure S12). Calcd. for $C_{66}H_{60}F_{12}Ir_2N_{10}O_2P_2$: C 46.64 H 3.56, N 8.24. Found C 46.60, H 3.55, N 8.22.

Ir-S-S-Ir was purified by silica gel column chromatography with dichloromethane/methanol (80/1 v/v) as eluting solvent. The product was a red solid with a yield of 80%. ^{1}H NMR (500 MHz, DMSO-*d$_6$*) δ 10.77 (s, 2H), 8.68 (s, 1H), 8.53 (d, *J* = 8.1 Hz, 2H), 8.28 (t, *J* = 8.4 Hz, 2H), 8.23 (d, *J* = 8.2 Hz, 2H), 8.17 (s, 2H), 7.95 (s, 4H), 7.89 (d, *J* = 7.7 Hz, 2H), 7.77 (dt, *J* = 12.9, 5.9 Hz, 6H), 7.45 (s, 2H), 7.27 (s, 2H), 7.20 (s, 2H), 6.99 (t, *J* = 7.4 Hz, 2H), 6.91 (t, *J* = 7.5 Hz, 2H), 6.84 (t, *J* = 7.8 Hz, 2H), 6.76 (q, *J* = 7.0 Hz, 2H), 6.18-6.13 (m, 2H), 6.02 (d, *J* = 7.4 Hz, 2H), 2.65 (s, 6H), 2.40-2.34 (m, 4H), 2.00 (s, 4H) (Figure S13). ^{13}C NMR (126 MHz, Methylene Chloride-*d$_2$*) δ 167.15, 166.92, 166.59, 154.61, 151.05, 150.82, 149.08, 148.79, 148.11, 143.88, 139.40, 138.72, 138.35, 132.22, 131.53, 130.73, 130.65, 130.47, 129.80, 128.68, 126.33, 124.89, 124.79, 123.91, 123.69, 123.37, 123.00, 119.71, 119.69, 33.17, 32.69, 31.93, 29.68, 17.67, 13.88 (Figure S14). ESI-MS: [m/z] = 724.1724 (M^{2+}) (calcd: 724.1724) (Figure S15). Calcd. for $C_{64}H_{56}F_{12}Ir_2N_{10}O_2P_2S_2$: C 44.29 H 3.25, N 8.07. Found C 44.30, H 3.27, N 8.10.

S1.2 Quantum chemical calculations

All calculations were performed in the Gaussian16 software package. The structures of B3LYP/6-31G*-D3 and the Hay-Wadt effective nuclear potential (ECP) of the iridium atoms and the basis set LANL2DZ were optimized to obtain a stable structure without virtual frequency. The frontier molecular orbital information including energy levels and distribution were obtained at the same theoretical level. Excited states were calculated in DMSO solution at the level of TD-B3LYP/6-31Gd, SMD, solvent = DMSO, and the photoelectric properties of the complexes were analyzed.

The energy transfer and charge transfer rates of the subject and guest doping models can be

calculated according to the semi-empirical Marcus charge transfer theory. The semi-empirical Marcus charge transfer theory is formulated as follows:

$$K = \left(\frac{4\pi^2}{h}\right) V_{HG}^2 \left(\frac{1}{\sqrt{4\pi\lambda k_B T}}\right) \exp\left[\frac{-(\Delta G+\lambda)^2}{4\lambda k_B T}\right]$$

Thus, the Gibbs free energy difference in the energy transfer process can be intuitively obtained according to the energy difference between the optimized $^1O_2$-S_0 and T_1-$^3O_2$ states.

S1.3 $^1O_2$ generation test with ABDA

The Ir complexes (20 μM) were mixed with ABDA (60 μM) solution as working solutions and then irradiated with 425 nm LED (20 mW cm^{-2}) for different times. The change of the absorption intensity of the indicator at 378 nm with time was monitored.

The ROS quantum yield of the Ir complexes in water (Φ) upon blue light irradiation (425 nm, 20 mW cm^{-2}) was determined using methylene blue (MB) as the standard reference. The ROS yield was calculated using the following equation:

$$\Phi_{\text{Ir Complex}} = \Phi_{\text{MB}} \left(K_{\text{Ir Complex}} \cdot A_{\text{MB}}\right) / K_{\text{MB}} \cdot A_{\text{Ir Complex}}$$

$K_{Ir\ Complex}$ and K_{MB} are the decomposition rate constants of the photosensitizing process determined by the plot ln(A_0/A) versus irradiation time. $A_{Ir\ Complex}$ and A_{MB} represent the light absorbed by the Ir complexes and MB, which are determined by the absorption at the wavelength of 425 nm. Φ_{MB} is the ROS quantum yield of MB, which is 0.52 in water.

S1.4 Test method for GSH consumption capacity in solution

S1.4.1 Test method for the structure change of Ir complexes after reacted with GSH

The Ir complexes (30 μM) were mixed with GSH (2 mM) solution as working solutions. The UV-visible absorption spectra of the solutions after different times (0, 0.5, 1, 3, 5, 10, 15, 20, 30, 40, 60 min) were measured.

Ir-S-S-Ir (0.71 mM) was mixed with different equivalents (3, 5, 10 and 20 eq) of GSH for 2 h as working solutions. HPLC analysis was performed on an Agilent Technologies 1200 Series instrument equipped with a TC-C18 column. Mobile Phase: methanol/water, 60/40 v/v; Flow rate: 1.0 mL/min; Temperature: 25 °C; Detection wavelength: 380 nm.

S1.4.2 Test method for GSH capacity of complex consumption

Ir-S-S-Ir with concentrations of 0, 50, 100, 150, 200, 250 μM was mixed with GSH solution (1.3 mM) as samples of different concentrations and reacted at room temperature for 6 h in phosphate-buffered saline (PBS; pH 7.4). The same volume of reaction liquid was taken from each sample and mixed with DTNB solution (0.396 mg/mL), and reacted at room temperature for 10 min. The absorption peak at 412 nm was measured by UV-vis absorption spectroscopy. The absorption intensity of each sample was introduced into the regression equation which was measured before to calculate the remaining GSH concentration after the reaction, and the proportion of GSH consumption was calculated.

S1.5 Cell culture

4T1 cells were maintained in RPMI 1640 growth medium supplemented with 10% serum and 1% penicillin and streptomycin. Cells were cultured at 37 °C in a humidified atmosphere of 95% air and 5% CO_2.

S1.6 Cytotoxicity test method

Cell dark/phototoxicity was detected by CCK-8 in 4T1 cells. 4T1 cells (8×10^3) were inoculated in 96-well plates under dark conditions for 12 hours. Following established protocols,[3-6] stock solutions of the complexes were prepared by dissolving the complexes in aqueous solutions with DMSO as the co-solvent and diluting with cell culture medium to the desired concentrations. The final DMSO

concentration never exceeded 1% v/v. After the fresh medium was replaced, Ir complexes with different concentrations (0, 2, 5, 10, 15, 20 μM) and cells were incubated for 12 hours. The cells were irradiated with white light (400-800 nm, 20 mW cm^{-2}) for 20 min. Under the same conditions, the dark control group did not receive any light treatment. After the two different treatments, the cells continued to be cultured in dark conditions for 12 hours. Then the old medium was removed, 10% CCK-8 dye diluted in fresh medium was added, and the survival rate of the living cells was calculated by testing the absorbance at 450 nm to evaluate the dark/phototoxicity of the material.

S1.7 Live & dead staining test method

4T1 cells were cultured for 24 h, then stained with Ir complexes for another 12 h. Later, the cells were divided into two groups. For dark groups, cells were kept under dark for another 12 h, and for light irradiation groups, cells were exposed to blue light (425 nm, 20 mW cm^{-2}) for 20 min and then incubated for another 12 h. After that, cells were incubated with Calcein-AM/PI for 30 min. For dark groups, cells were kept under dark, and for light irradiation groups, cells were exposed to white light (400-800 nm, 20 mW cm^{-2}) for 20 min. Green channel: λ_{ex} = 488 nm, λ_{em} = 500-550 nm. Red channel: λ_{ex} = 543 nm, λ_{em} = 600-700 nm. Scale bar: 50 μm.

S1.8 Evaluation of intracellular ROS production capacity

4T1 cells were cultured for 24 h, then stained with Ir complexes for another 12 h. Later, the incubation medium was discarded, and cells were washed with PBS 3 times. After that, cells were incubated with FBS-free media containing DCFH-DA (1 μM) for 30 min. For dark groups, cells were kept under dark conditions, and for light irradiation groups, cells were exposed to white light (400-800 nm, 20 mW cm^{-2}) for 20 min. Green channel: λ_{ex} = 488 nm, λ_{em} = 500-550 nm. Scale bar: 50 μm.

S1.9 Test method for intracellular GSH content

The reduced glutathione (GSH) content detection kit was used to detect the decrease of GSH content in 4T1 cells after **Ir-S-S-Ir** incubation. 4T1 cells were cultured in six-well plates for 24 h, and then incubated with **Ir-S-S-Ir** of different concentrations (0, 20, 40, 60, 80 μM) for 24 h. The cultured 4T1 cells were collected and washed twice with PBS. The instructions for the reduced glutathione (GSH) content test kit were followed.

S1.10 Test method for cell uptake capacity

4T1 cells were inoculated into confocal culture dishes at a density of 5×10^4 cells per well, and then incubated in RPMI 1640 medium containing 10% fetal bovine serum under standard cell culture conditions of 5% CO_2 at 37 °C for 24 h. After incubation, the old medium was sucked out and fresh medium containing Ir complexes (20 μM) was added to the confocal dish. After incubation for 1, 6, 12 and 24 h, the original medium was sucked out, 4T1 cells were washed three times with PBS, and fresh medium was added. The uptake of Ir complexes by 4T1 cells at different incubation times was observed by a confocal laser scanning microscope.

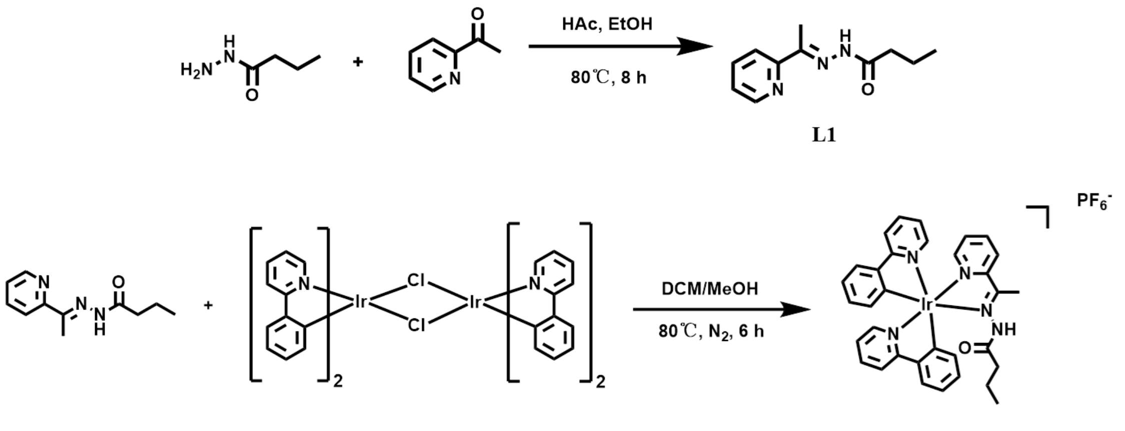

L1

Ir-C

Fig. S1. Synthetic procedure for the preparation of **Ir-C**.

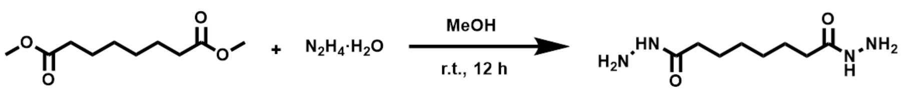

1

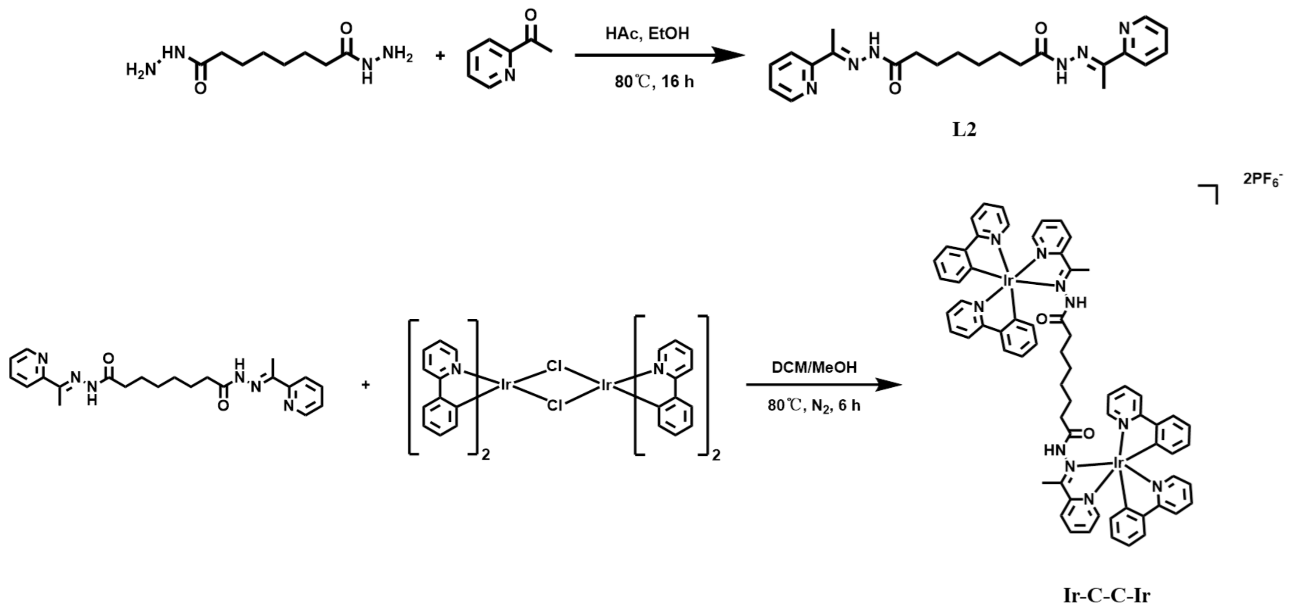

Fig. S2. Synthetic procedure for the preparation of **Ir-C-C-Ir**.

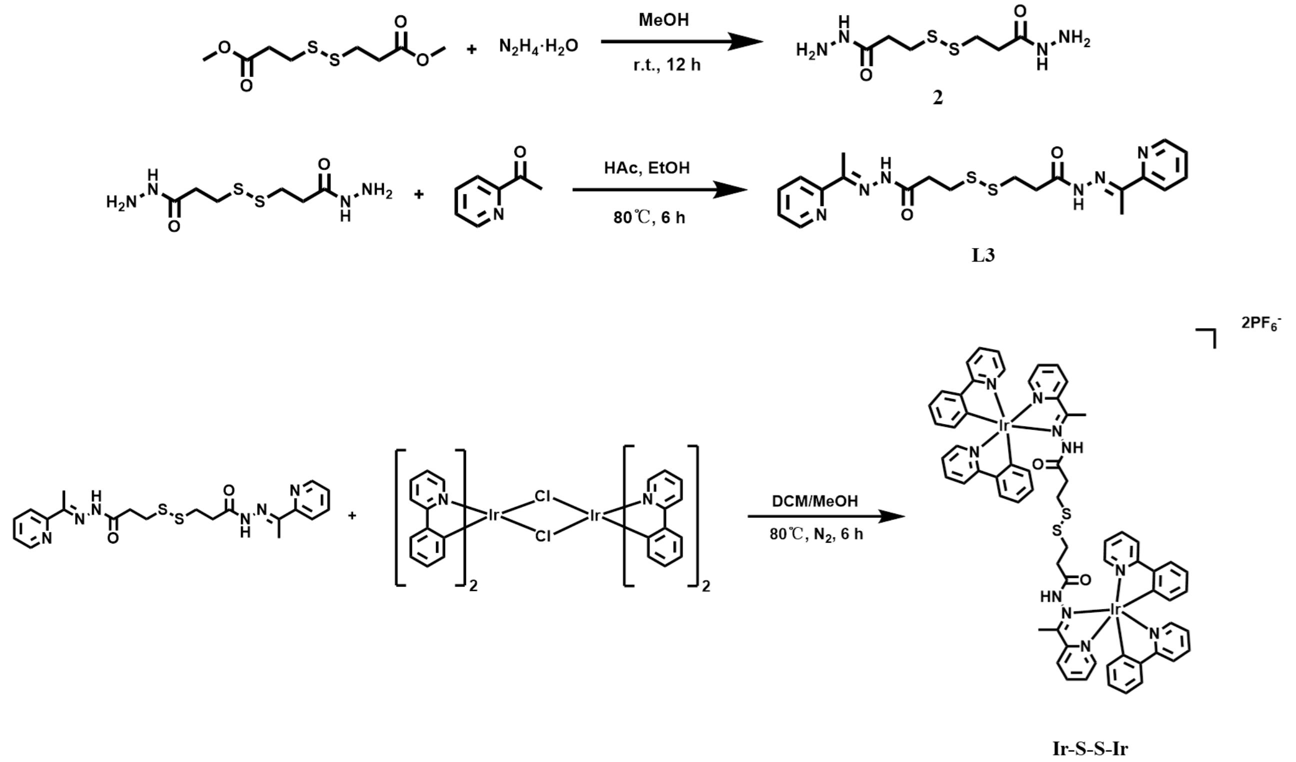

Fig. S3. Synthetic procedure for the preparation of **Ir-S-S-Ir**.

S1.2 Characterization

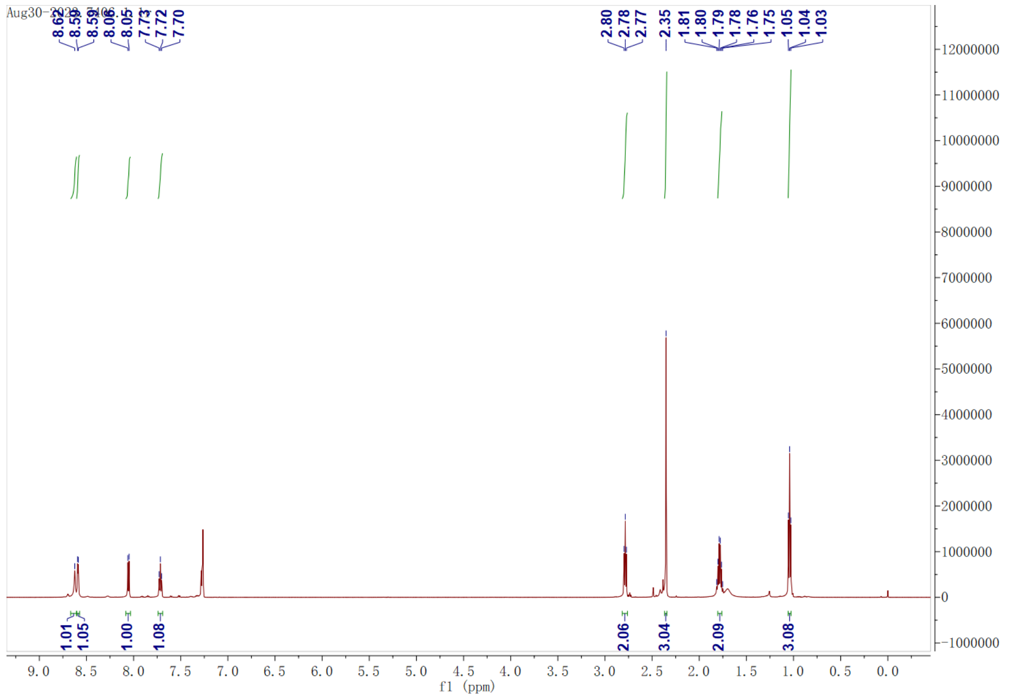

Fig. S4. ^{1}H NMR spectrum of **L1** in $CDCl_3$.

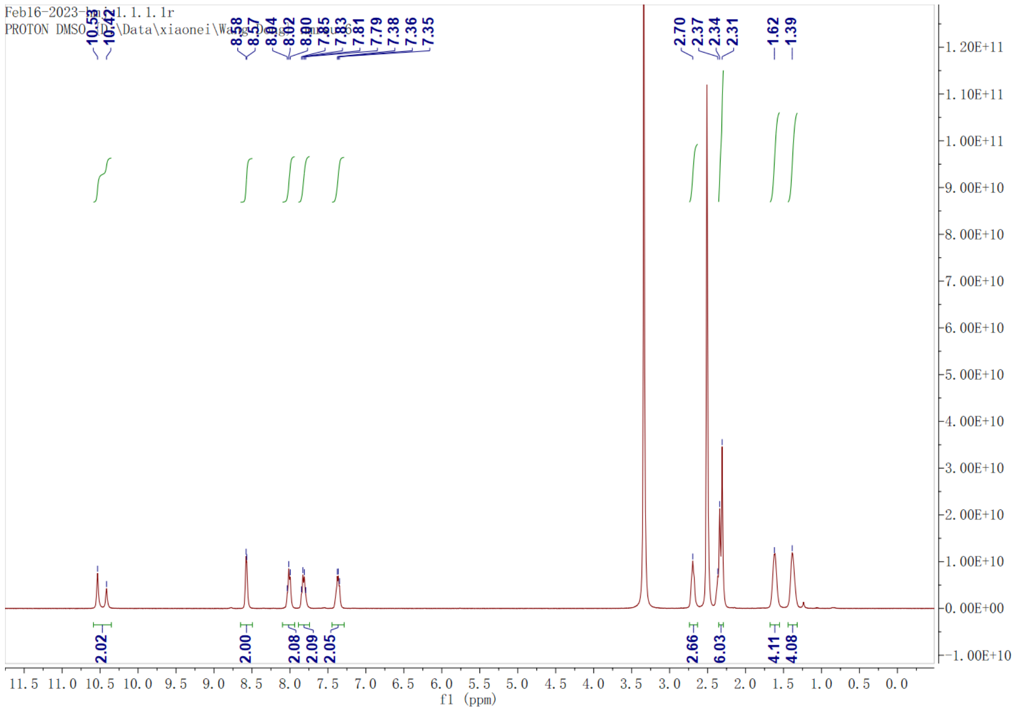

Fig. S5. ^{1}H NMR spectrum of **L2** in DMSO-d_6.

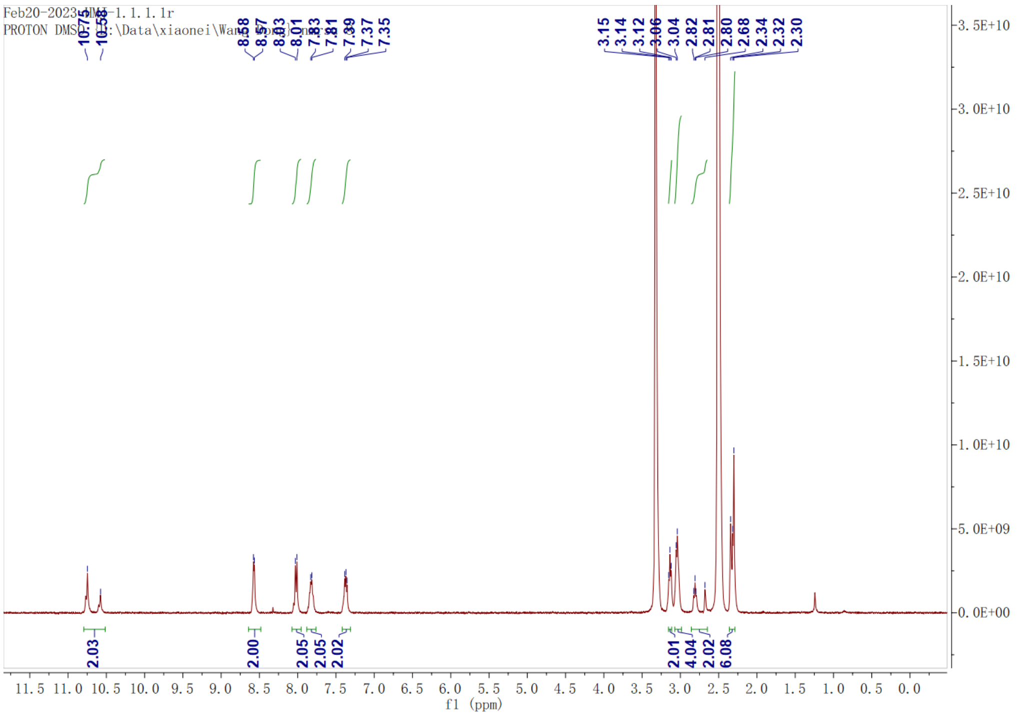

Fig. S6. ^{1}H NMR spectrum of **L3** in DMSO-d_6.

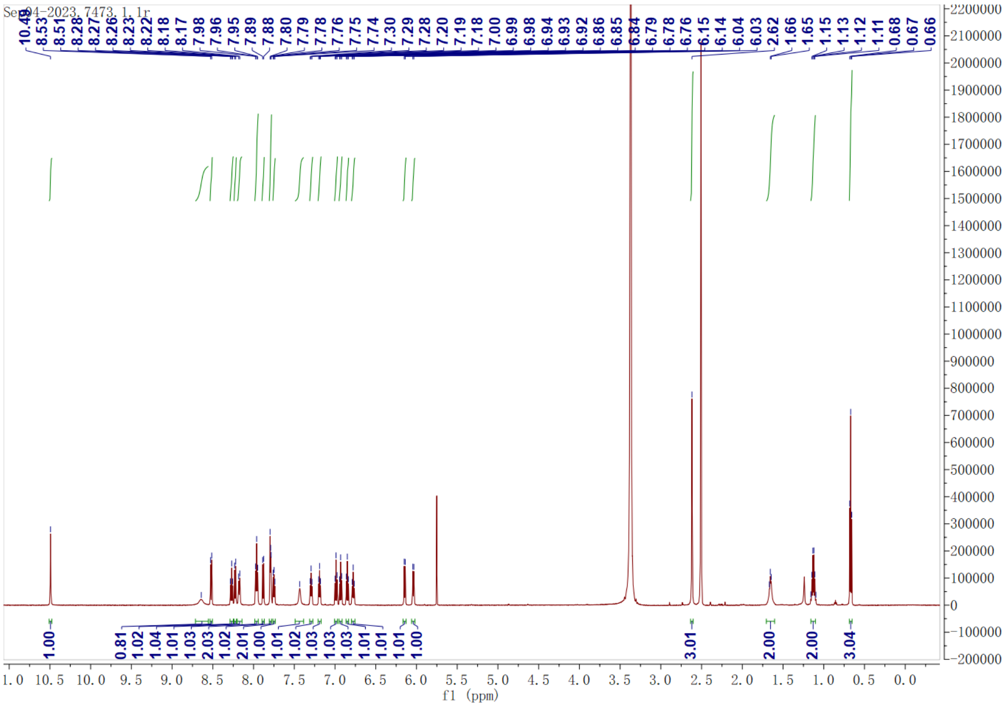

Fig. S7. ^{1}H NMR spectrum of **Ir-C** in DMSO-d_6.

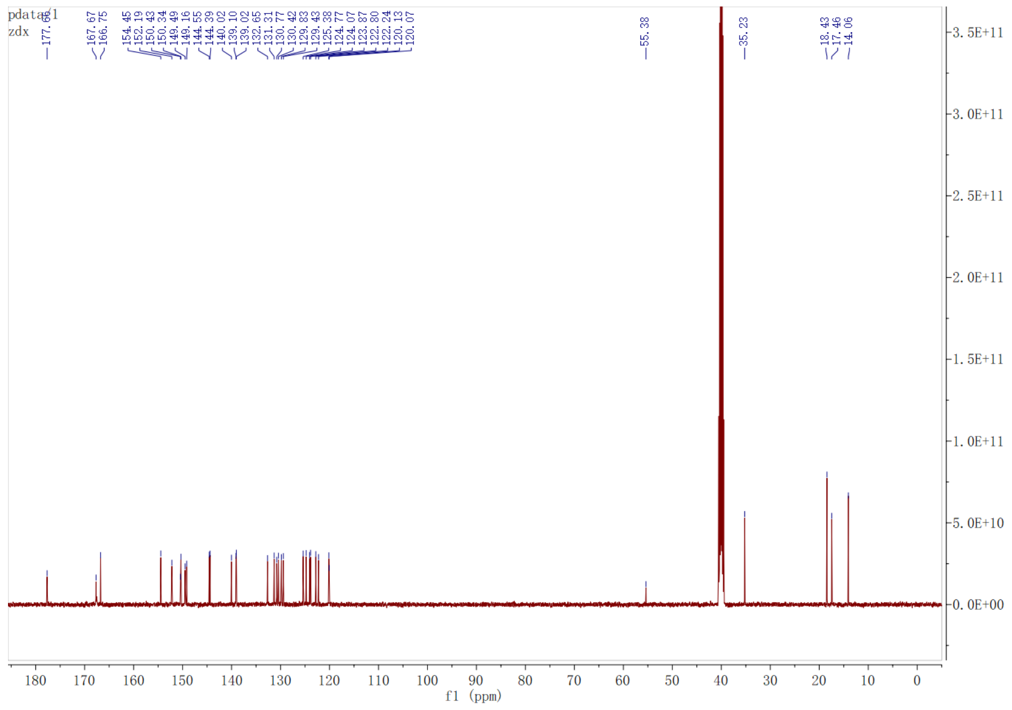

Fig. S8. ^{13}C NMR spectrum of **Ir-C** in DMSO-d_6.

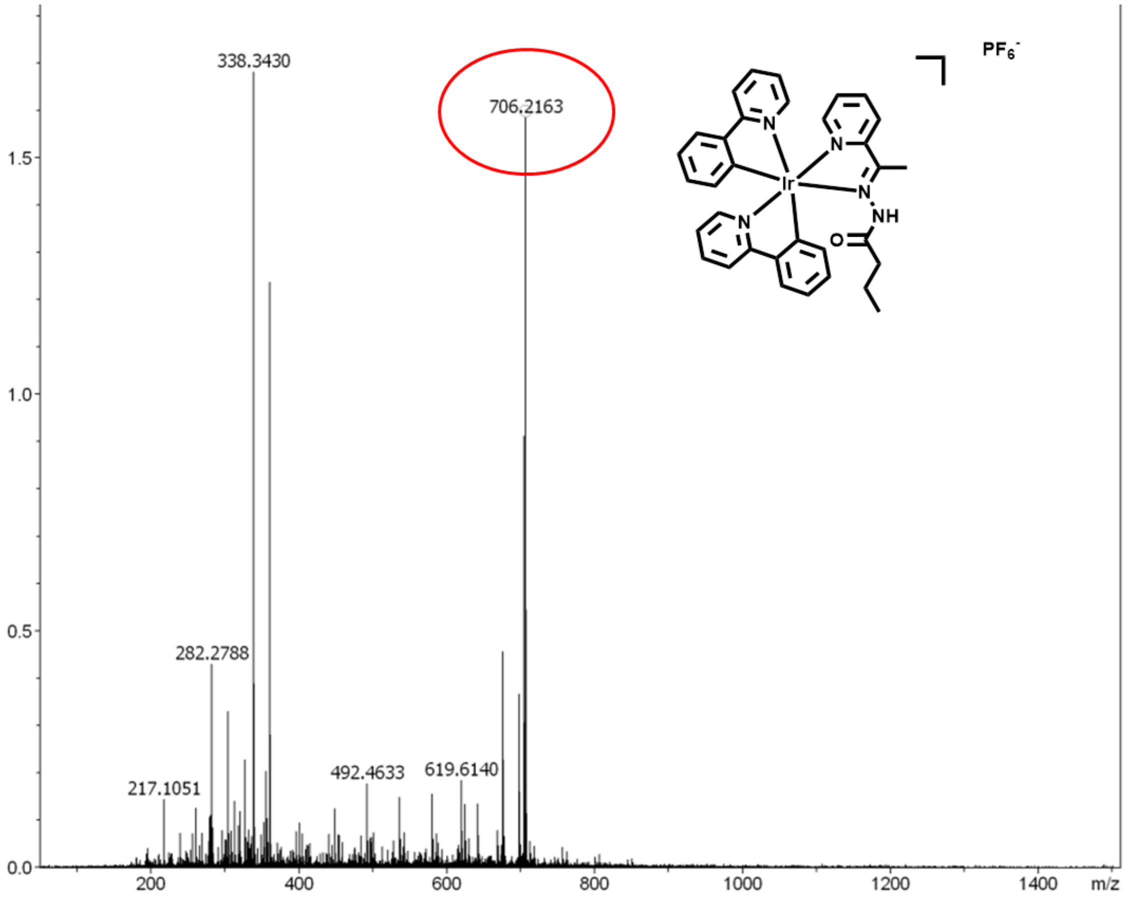

Fig. S9. Mass spectrum of **Ir-C**.

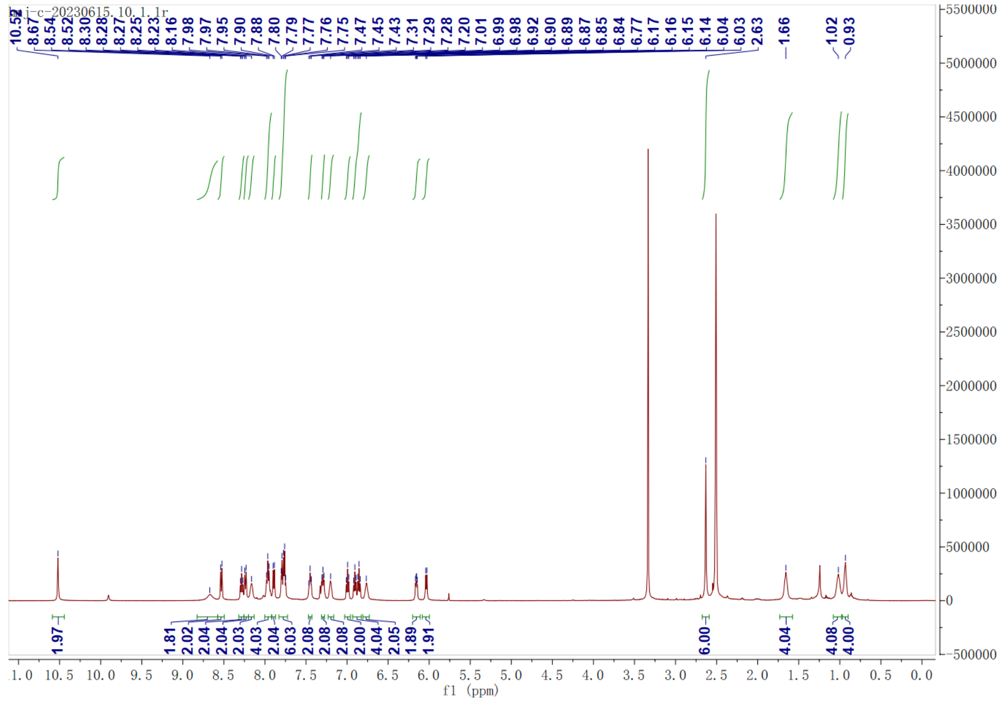

Fig. S10. ^{1}H NMR spectrum of **Ir-C-C-Ir** in DMSO-d_6.

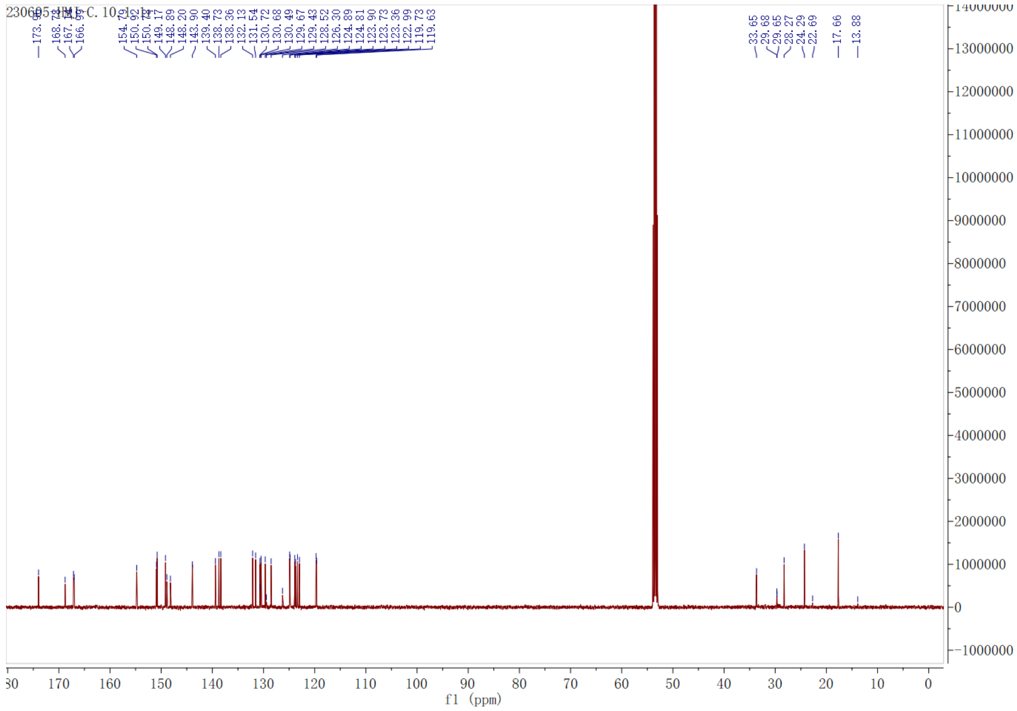

Fig. S11. ^{13}C NMR spectrum of **Ir-C-C-Ir** in CD_2Cl_2.

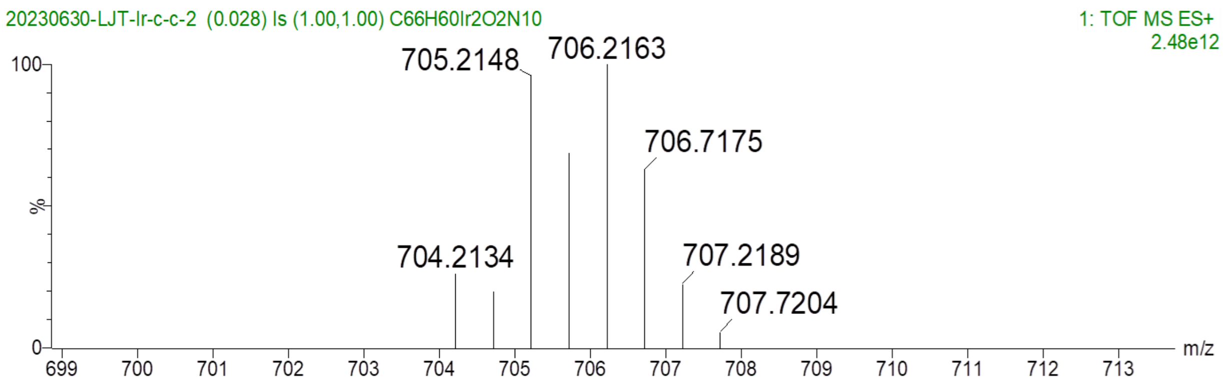

Fig. S12. Mass spectrum of **Ir-C-C-Ir**.

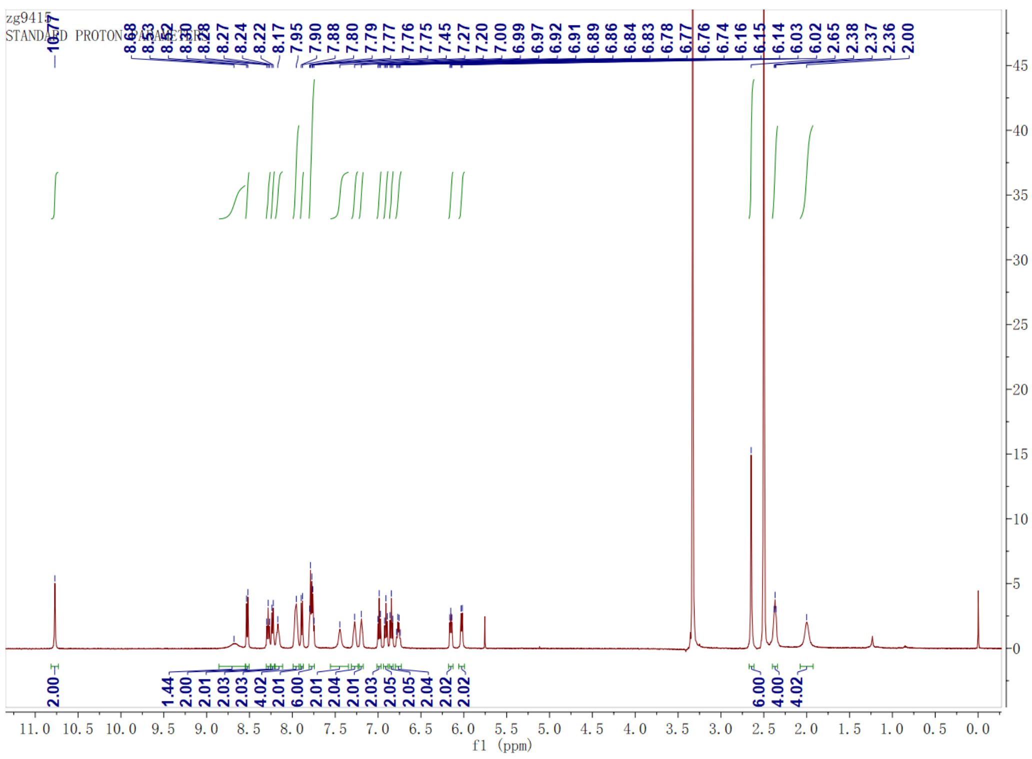

Fig. S13. ^{1}H NMR spectrum of **Ir-S-S-Ir** in DMSO-d_6.

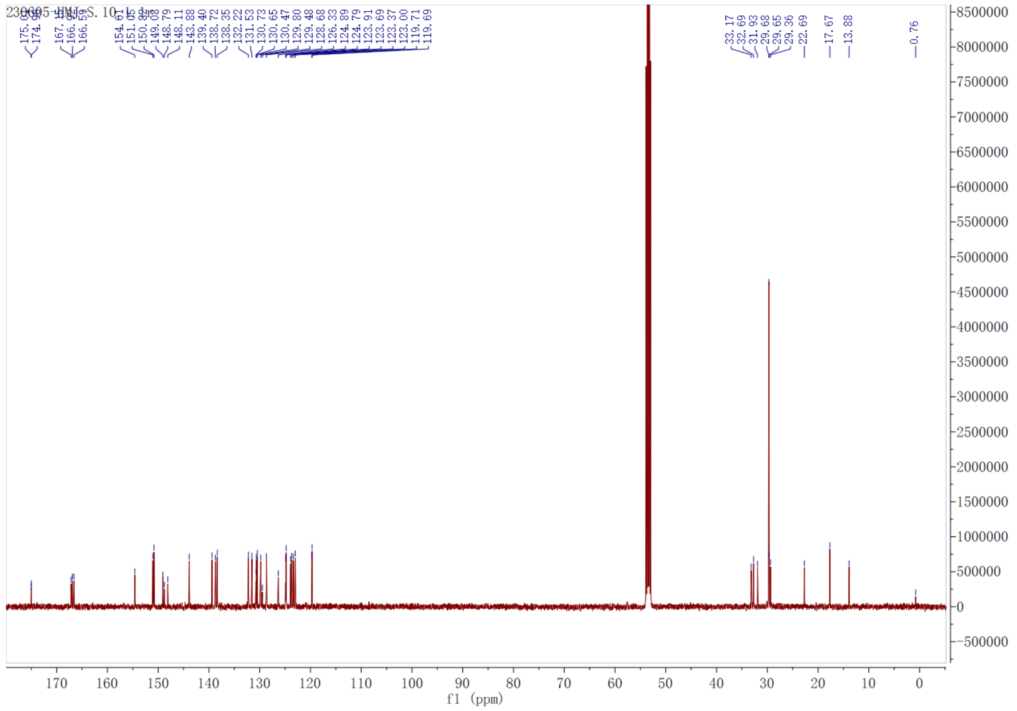

Fig. S14. ^{13}C NMR spectrum of **Ir-S-S-Ir** in CD_2Cl_2.

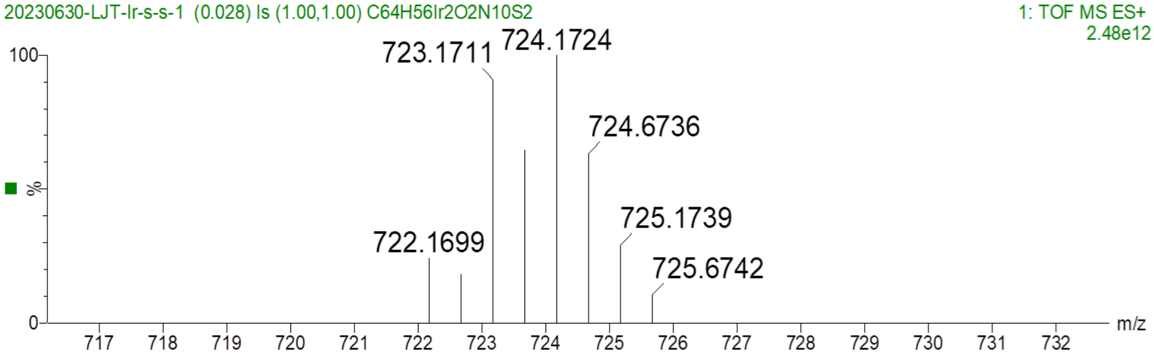

Fig. S15. Mass spectrum of **Ir-S-S-Ir**.

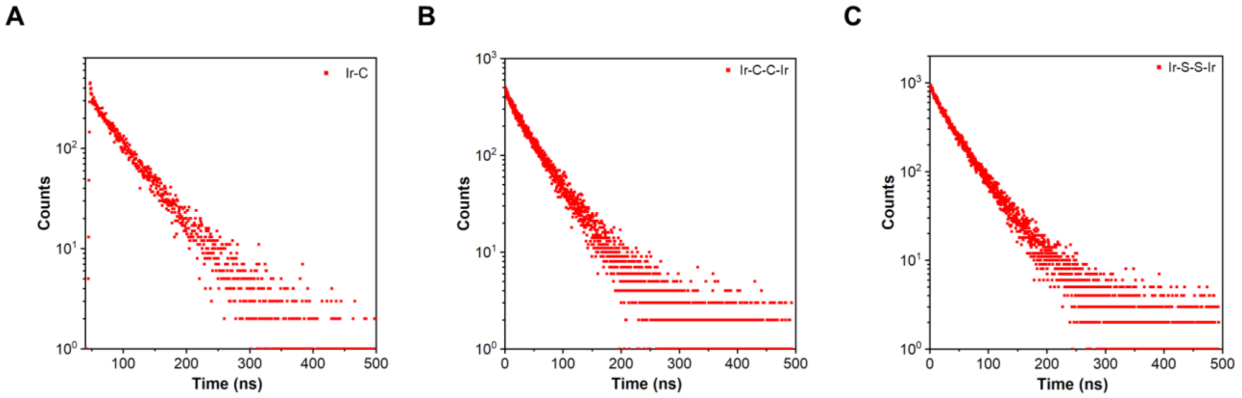

Fig. S16. Phosphorescence lifetime of (A) **Ir-C**, (B) **Ir-C-C-Ir,** (C) **Ir-S-S-Ir** in solid state.

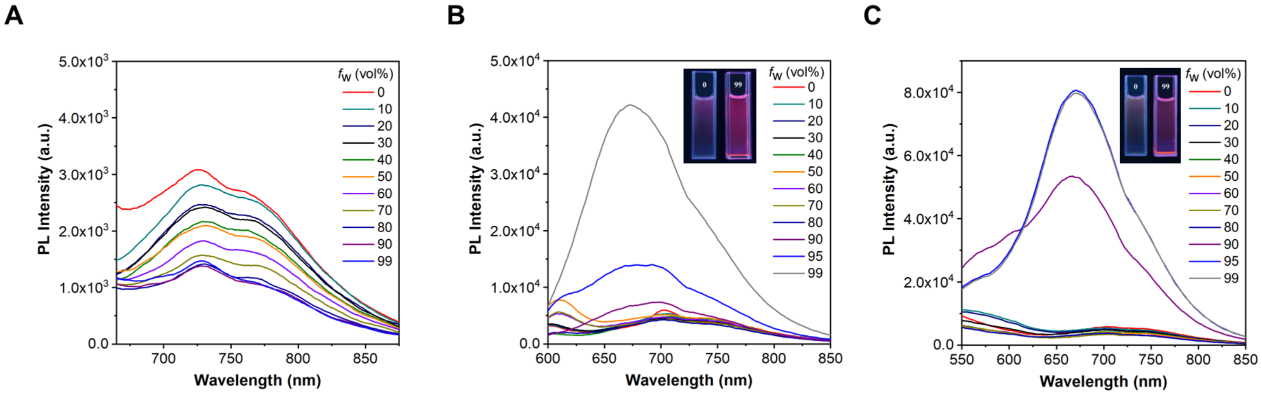

Fig. S17. Emission spectra of (A) **Ir-C**, (B) **Ir-C-C-Ir**, (C) **Ir-S-S-Ir** (5×10^{-5} M) in CH_3CN/PBS mixture, λ_{ex} = 380 nm, pH 7.4.

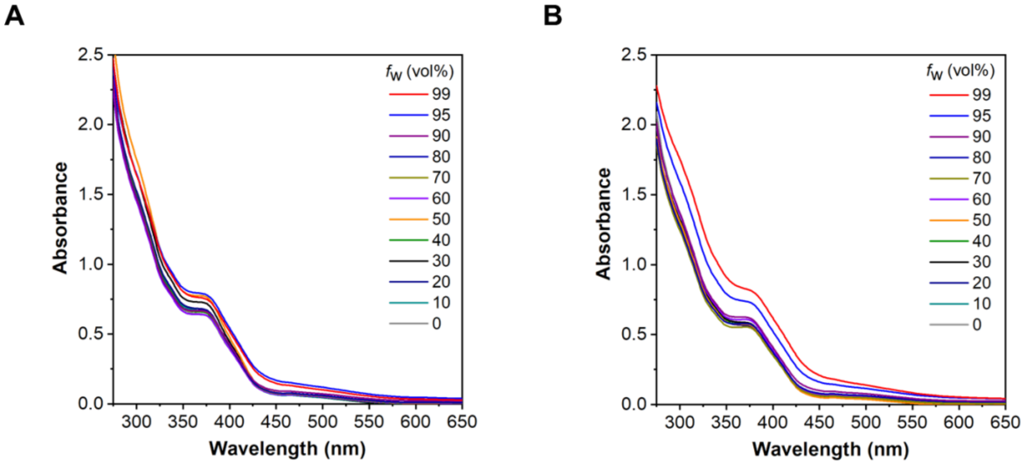

Fig. S18. UV-vis absorption spectra of (A) **Ir-C-C-Ir** and (B) **Ir-S-S-Ir** (5×10^{-5} M) in CH_3CN/H_2O

mixtures with different water fractions (0–99%, v/v) at room temperature.

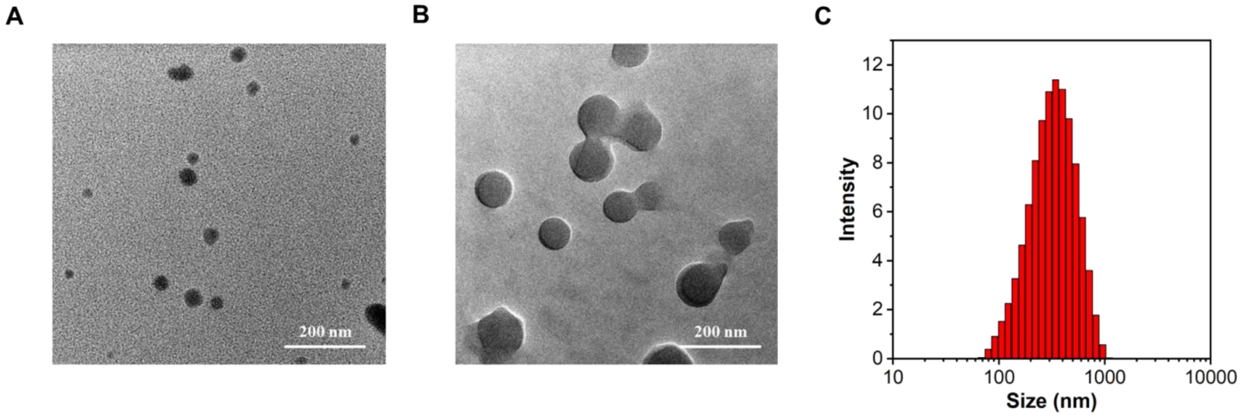

Fig. S19. TEM image of nanoaggregates of **Ir-C-C-Ir** formed in CH_3CN-H_2O mixtures with 0% water fraction (A) and 99% (B) water fraction. (C) Size distributions of nanoaggregates of **Ir-C-C-Ir** formed in CH_3CN/H_2O mixtures with 99% water fraction measured by DLS.

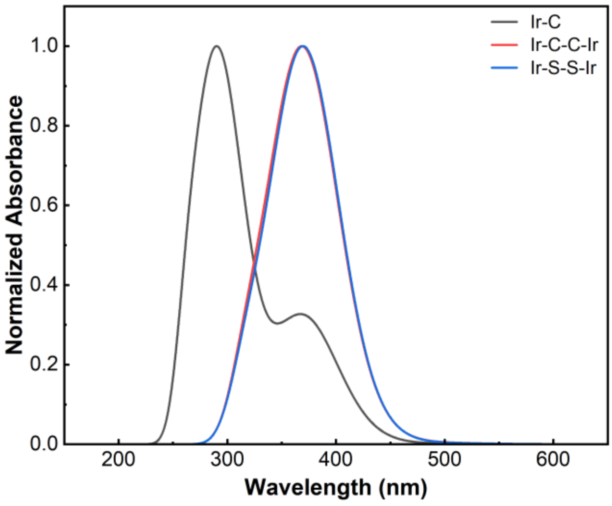

Fig. S20. Theoretical absorption spectra of **Ir-C**, **Ir-C-C-Ir** and **Ir-S-S-Ir** in water.

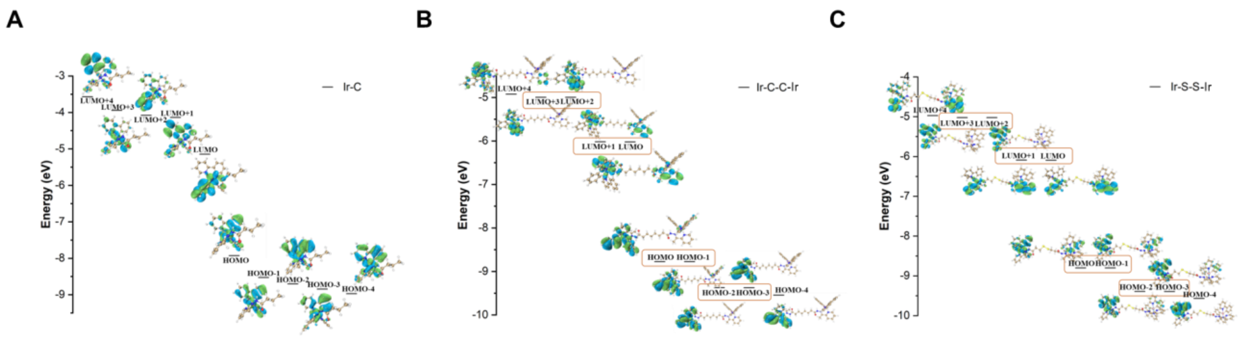

Fig. S21. (A) **Ir-C**, (B) **Ir-C-C-Ir** and (C) **Ir-S-S-Ir** LUMO+4 to HOMO-4 level distribution.

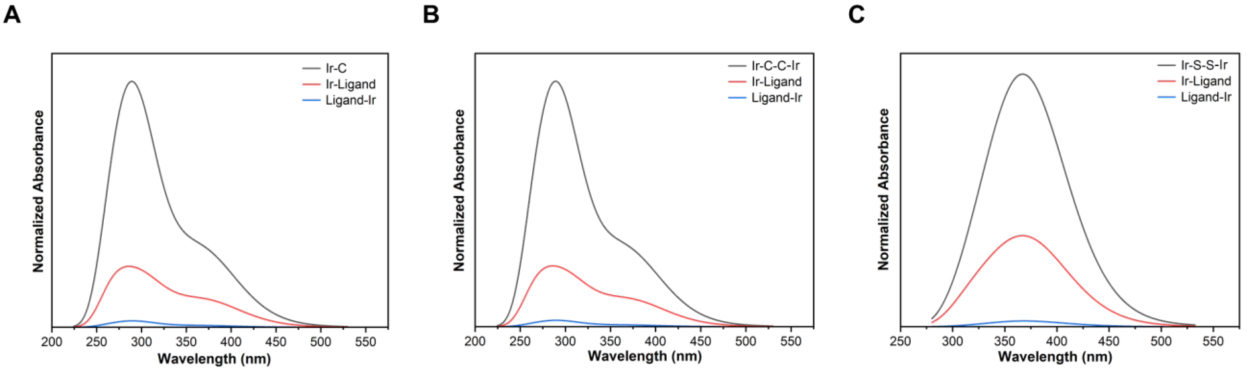

Fig. S22. Intramolecular charge transfer absorption spectrum of (A) **Ir-C**, (B) **Ir-C-C-Ir** and (C) **Ir-S-S-Ir**.

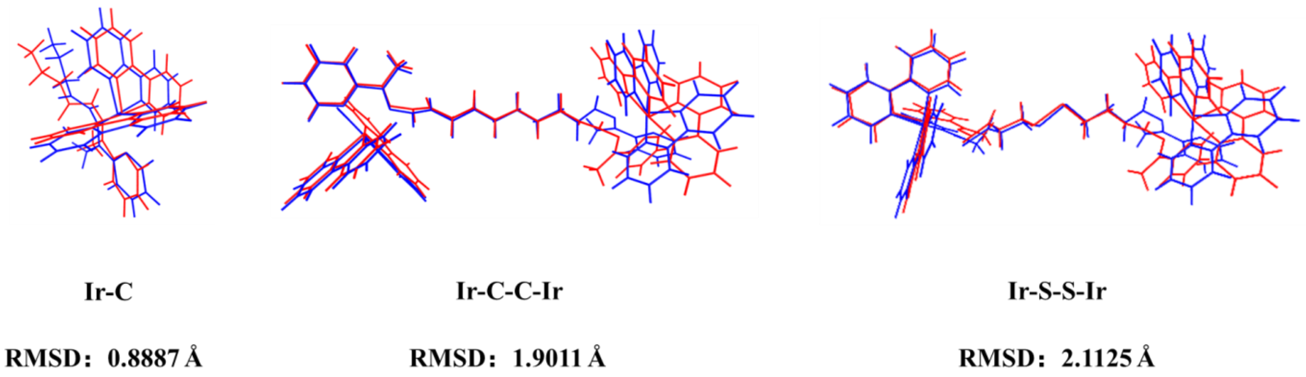

Fig. S23. Schematic diagram of S_0 and T_1 configurational changes and RMSD values of **Ir-C**, **Ir-C-C-Ir** and **Ir-S-S-Ir** after structural relaxation.

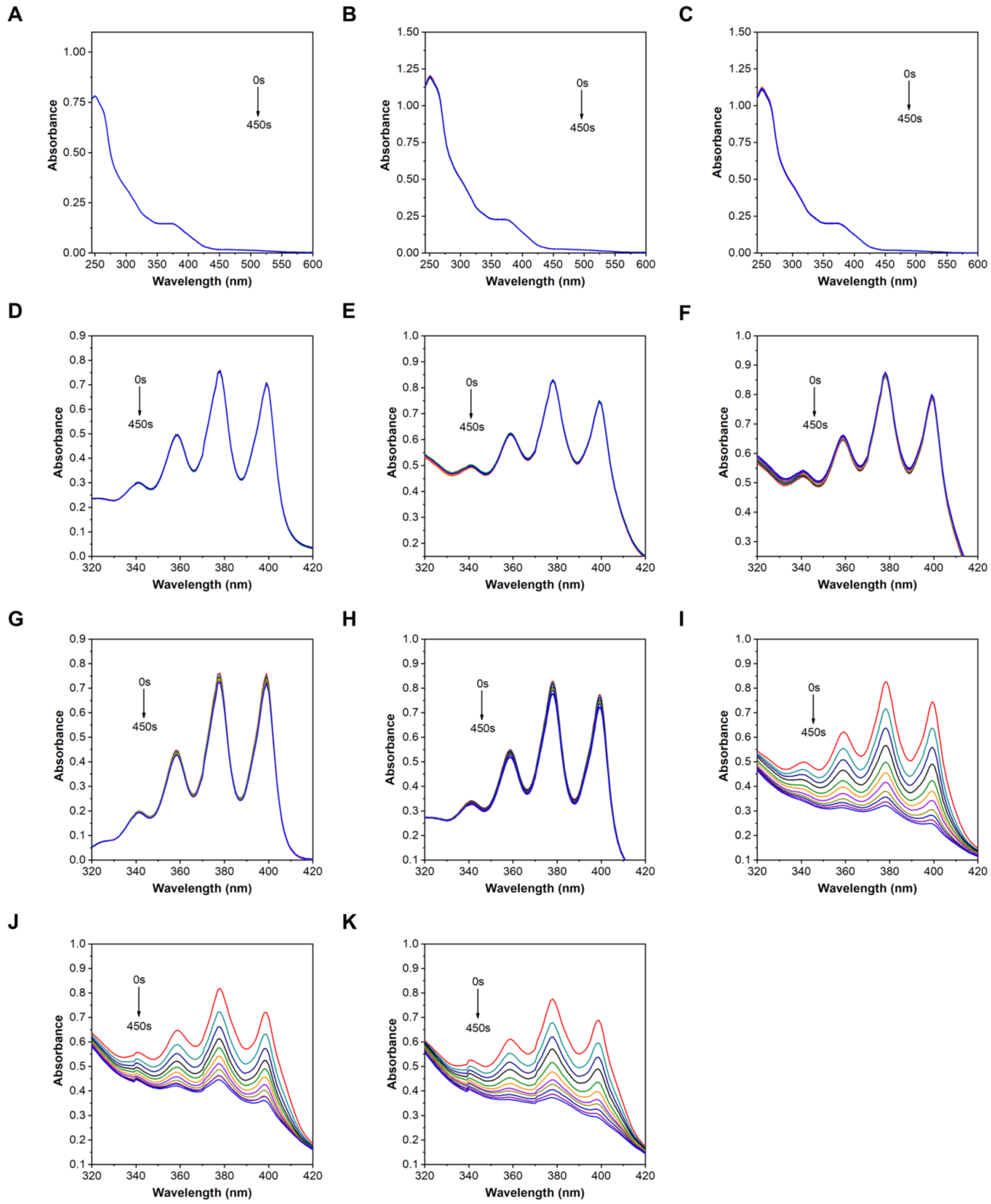

Fig. S24. UV-vis absorption spectra of (A) **Ir-C**, (B) **Ir-C-C-Ir** and (C) **Ir-S-S-Ir** under blue light (425 nm, 20 mW cm^{-2}). UV-vis absorption spectra of (D) **Ir-C**, (E) **Ir-C-C-Ir** and (F) **Ir-S-S-Ir** mixed with ABDA respectively in the dark. (G) UV-vis absorption spectra of ABDA under blue light (425 nm, 20 mW cm^{-2}). UV-vis absorption spectra of (H) **Ir-C** and (I) **Ir-C-C-Ir** mixed with ABDA respectively under blue light (425 nm, 20 mW cm^{-2}). UV-vis absorption spectra of (J) **Ir-C-C-Ir** and (K) **Ir-S-S-Ir** mixed with ABDA and GSH respectively under blue light (425 nm, 20 mW cm^{-2}).

Concentration: Ir complex (20 μM), ABDA (60 μM), GSH (10 mM).

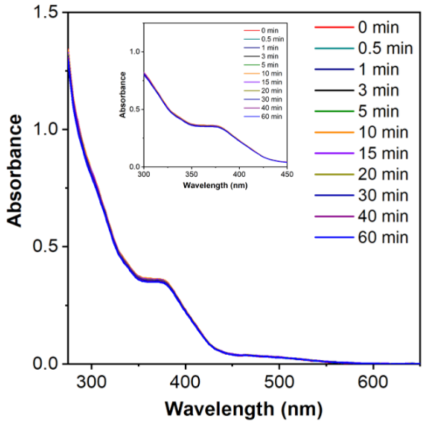

Fig. S25. UV-vis absorption spectra of **Ir-C-C-Ir** (30 μM) mixed with GSH (2 mM) in aqueous solution for different times. Inset: 300-450 nm region.

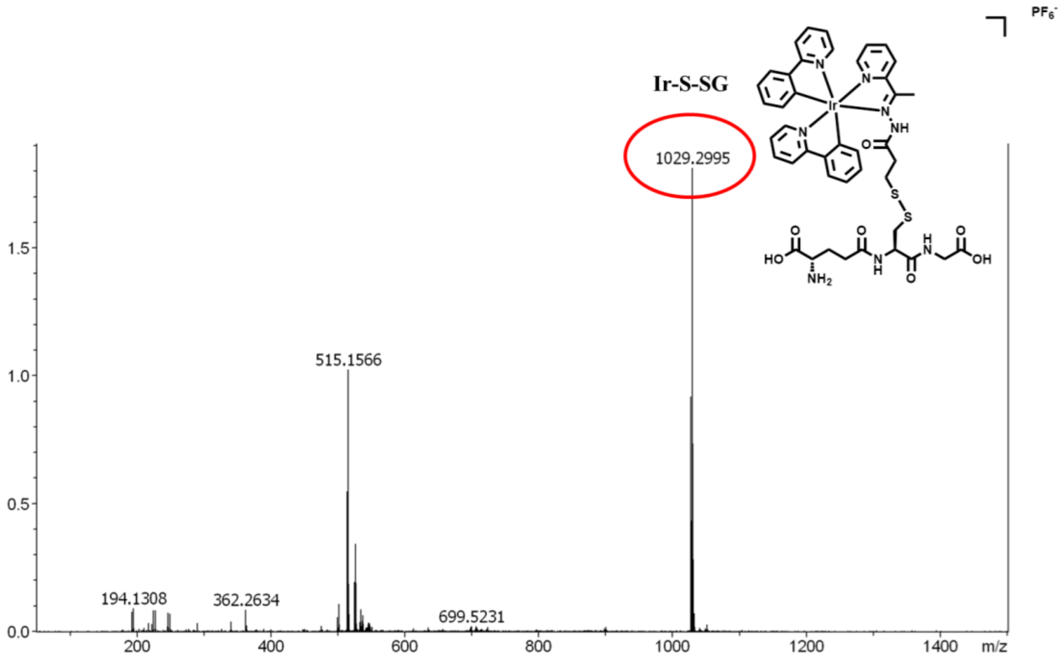

Fig. S26. Mass spectrum of **Ir-S-S-Ir** reaction with GSH.

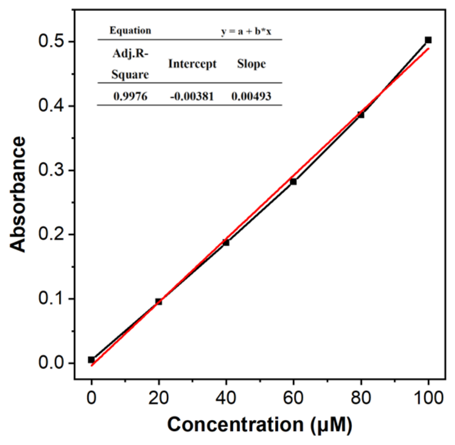

Fig. S27. Standard curve of absorbance versus GSH concentration.

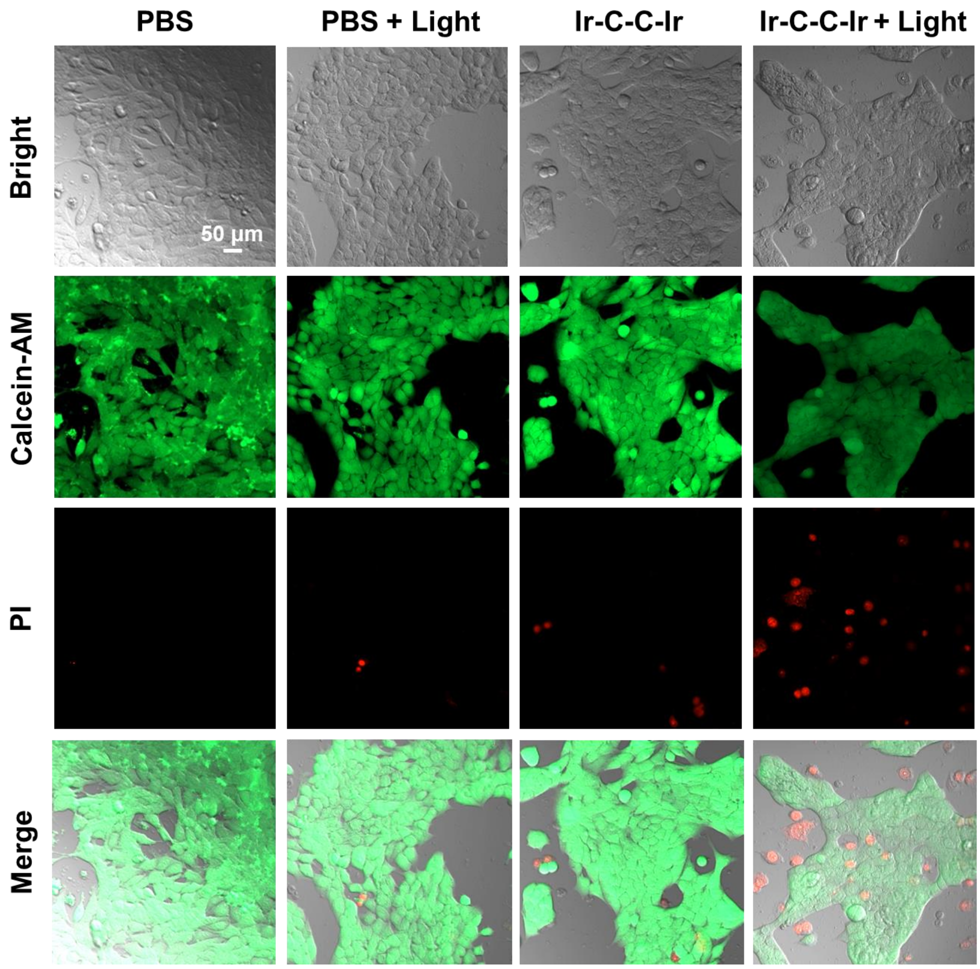

Fig. S28. Fluorescence images of living/dead 4T1 cells incubated with **Ir-C-C-Ir** (50 μM) under white light (400-800 nm, 20 mW cm^{-2}) and dark conditions.

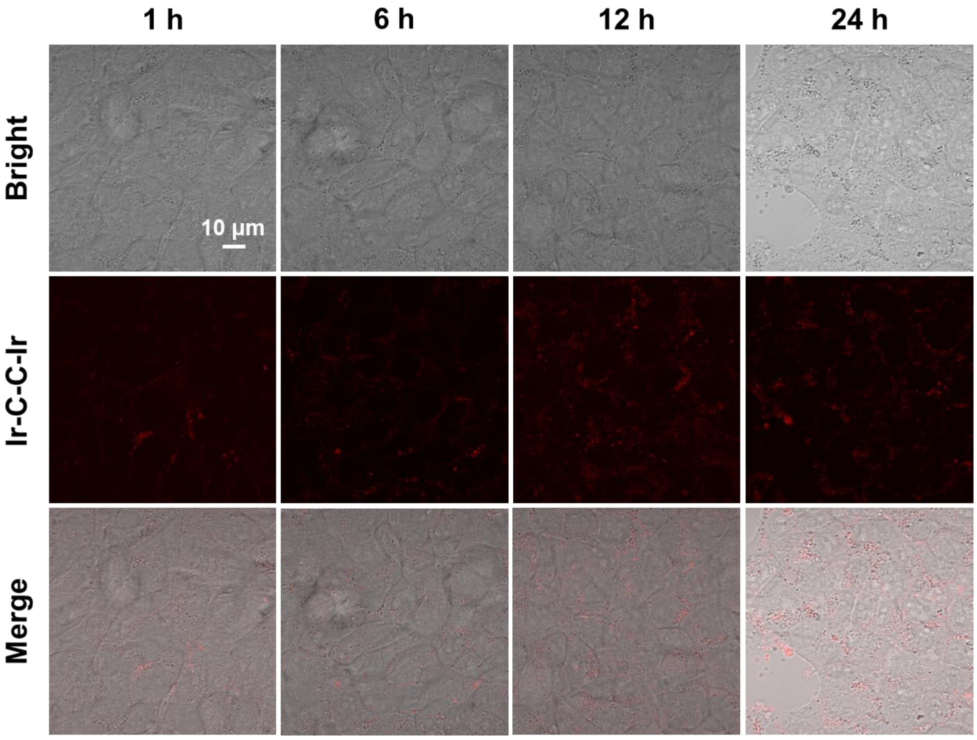

Fig. S29. CLSM images after **Ir-C-C-Ir** was incubated with 4T1 cells for 1 h, 6 h, 12 h and 24 h.

Table S1. Photophysical characteristics of **Ir-C**, **Ir-C-C-Ir** and **Ir-S-S-Ir**.

	λ_{abs}① (nm)	λ_{em}① (nm)	Φ_p② (%)	τ_p (ns) ②③	ε① ($\times10^3$ $M^{-1}cm^{-1}$)	k_r ($\times10^6$ s^{-1})	k_{nr} ($\times10^6$ s^{-1})
**Ir-C**	258; 374	720	1.30	51.9	60.3; 6.7	0.25	19.02
**Ir-C-C-Ir**	254; 377	719	1.40	38.2	64.6; 11.9	0.36	25.82
**Ir-S-S-Ir**	252; 377	720	1.20	43.1	65.3; 11.9	0.28	22.92

① Measured in CH_3CN (1.0×10^{-5} M) λ_{ex} = 380 nm; ② Measured in solid state, λ_{ex} = 380 nm; ③ Determined in normal oxygen environment; ④ The radiative k_r and k_{nr} in solid-state were calculated by: $k_r = \Phi/\tau$ and $k_{nr} = (1-\Phi)/\tau$.

Table S2. The excited state properties of the complexes at the level of TD-B3LYP/6-31G(d) SMD, DMSO

	Configuration	Excited energy/eV	Oscillator strength	Wavelength/nm	Major transition components	Ir-ligand |q|/e
**Ir-C**	S_1	2.532	0.0009	489.67	H -> L 98.7%	0.3742
	S_6	3.488	0.0943	355.45	H-3 -> L 76.1%	0.3231
**Ir-C-C-Ir**	S_1	2.536	0.0007	488.88	H-1 -> L 69.0%	0.374
	S_5/S_6	3.176/3.176	0.0366/0.0797	390.42/390.35	H -> L+3 40.6%/ H-1 -> L+2 39.6%	0.39/0.394
**Ir-S-S-Ir**	S_1	2.518	0.0006	492.41	H -> L 54.8%	0.3731
	S_7/S_8	3.179/3.180	0.0506/0.0650	390.05/389.90	H -> L+3 74.3%/ H-1 -> L+2 65.9%	0.3919/0.4025

Table S3. Time-dependent $^1O_2$ generation kinetics data under different conditions.

**Equation**		**y = a + b*x**		
	**Adj.R-Square**	**Intercept**	**Slope**	**$^1O_2$ quantum yield (%)**
**Ir-S-S-Ir**	0.98565	0.06196	0.00238	76.7
**Ir-C-C-Ir**	0.97813	0.07187	0.00211	31.5
**Ir-C**	0.92613	0.00257	0.00013	2.3
MB	0.99440	-0.00218	0.00025	52.0
**Ir-S-S-Ir** + GSH	0.97048	0.07407	0.00160	46.9
**Ir-C-C-Ir** + GSH	0.95453	0.08071	0.00131	15.7
ABDA	0.95772	0.00552	0.00011	-

References

1. Nonoyama, M. *Bull. Chem. Soc. Jpn*. **1974**, *47*, 767–768.
2. Sprouse, S.; King, K. A.; Spellane, P. J.; Watts, R. J. *J. Am. Chem. Soc.* **1984**, *106* (22), 6647–6653.
3. Redrado, M.; Benedi, A.; Marzo, I.; Gimeno, M. C.; Fernández-Moreira, V. Dual Emissive Ir(III) Complexes for Photodynamic Therapy and Bioimaging. *Pharmaceutics* **2021**, *13* (9), 1382.
4. Gonzalo-Navarro, C.; Zafon, E.; Organero, J. A.; Jalón, F. A.; Lima, J. C.; Espino, G.; Rodríguez, A. M.; Santos, L.; Moro, A. J.; Barrabés, S.; Castro, J.; Camacho-Aguayo, J.;

Massaguer, A.; Manzano, B. R.; Durá, G., Ir(III) Half-Sandwich Photosensitizers with a π-Expansive Ligand for Efficient Anticancer Photodynamic Therapy. *Journal of Medicinal Chemistry* **2024**, *67* (3), 1783-1811.

5. Tabrizi, L.; Chiniforoshan, H., New cyclometalated Ir(III) complexes with NCN pincer and meso-phenylcyanamide BODIPY ligands as efficient photodynamic therapy agents. *RSC Advances* **2017**, *7* (54), 34160-34169.
6. Ding, R.; Liu, X.; Zhang, W.; Chen, X.; Chen, S.; Yu, X.; Zhao, Z.; Li, K., Purine-Based Ir(III) Photosensitizers for Efficient Treatment of Periodontal Inflammation. *Advanced Functional Materials* **2024**, 2405499.
